# Detection of Antagonistic Interactions Between Cannabidiol and Temozolomide in Various Neuroblastoma and Glioblastoma Cell Lines in the MTT Assay by Means of Synergy Finder 3.0 Software and Log-Probit Isobolographic Analysis

**DOI:** 10.3390/ijms27219946

**Published:** 2026-11-06

**Authors:** Katarzyna Załuska-Ogryzek, Paula Wróblewska-Łuczka, Agnieszka Góralczyk, Jarogniew J. Łuszczki

**Affiliations:** Department of Occupational Medicine, Medical University of Lublin, 20-090 Lublin, Poland; katarzyna.zaluska-ogryzek@umlub.edu.pl (K.Z.-O.); paula.wroblewska-luczka@umlub.edu.pl (P.W.-Ł.); agnieszka.goralczyk@umlub.edu.pl (A.G.)

**Keywords:** cannabidiol, temozolomide, drug interaction, isobolographic analysis, synergy finder, neuroblastoma, glioblastoma

## Abstract

Despite progress in experimental neuro-oncology and advances in the treatment of brain cancers, glioblastoma multiforme is still the most aggressive primary brain tumor in adults. Cannabinoid-based add-on therapy is likely to be a treatment option for glioblastoma patients who have failed surgical excision and treatment with temozolomide (a classic chemotherapeutic drug). Cannabidiol is one of the naturally occurring compounds isolated from Cannabis, which displays the anti-cancer and anti-viability effects in several glioblastoma cell lines. In the MTT assay, the anti-viability effects of combined treatment with cannabidiol and temozolomide were assessed in two neuroblastoma (CHP-134, KELLY) and four glioblastoma (SF295, U-87MG, T98G, and C6) cell lines. The interactions between cannabidiol and temozolomide from the MTT assay were characterized by means of two independent methods: a classic log-probit isobolographic analysis and a Synergy Finder 3.0 web-application allowing automatic evaluation of interactions in five pharmacodynamic models, including the zero interaction potency (ZIP), Bliss, Loewe, highest single agent (HAS) and Bliss–Loewe consensus models. The impact of cannabidiol on several apoptotic-related proteins (p21, p18, cyclin D1, Bax, Bcl-2) and the cannabinoid CB_1_ receptor expressed on the glioblastoma SF295 and U-87MG cells was examined with the Western blot technique. Both cell migration and cell–cell interaction after treatment with cannabidiol, temozolomide and their combination were assessed in a wound healing assay. The log-probit isobolographic analysis of interactions revealed that cannabidiol combined with temozolomide exerted antagonistic interactions only in two neuroblastoma cell lines, CHP-134 and KELLY. In contrast, the combination of cannabidiol with temozolomide in all the glioblastoma cells, SF295, U-87MG, T98 and C6, produced (in the classic isobolography) additive interactions with a tendency toward antagonism in the MTT assay. With Synergy Finder 3.0 in the Bliss–Loewe model, the interactions between cannabidiol and temozolomide were antagonistic in all the tested cell lines (except for CHP-134) with synergy score values ranging from −19.64 (for T98G) to −15.07 (for U-87MG). Only the interaction between cannabidiol and temozolomide in the CHP-134 cell line was additive in the Bliss–Loewe model with a synergy score value of 0.95. This comparative study indicates that the combination of cannabidiol with temozolomide should not be recommended and used clinically because of antagonistic interactions reported in the MTT assay. The Bliss–Loewe consensus model can be recommended as a model of choice when screening and evaluating the types of interactions in in vitro studies using the Synergy Finder 3.0 free online available web-application.

## 1. Introduction

Glioblastoma is one of the most malignant brain cancers in humans [1,2]. Patients with glioblastoma underwent aggressive treatment consisting usually of three consecutive steps: (a) surgical excision of the cancerous tissue (if possible); (b) chemotherapy with temozolomide (a chemotherapeutic drug approved for the treatment of glioblastoma patients); (c) radiotherapy to reduce reoccurrence of glioblastoma [1,2]. Despite such aggressive treatment, the mean survival rate is low, amounting to 13.5 months [3,4]. A poor prognosis in glioblastoma patients obligates researchers to search for some novel drugs or compounds offering efficacious anti-glioblastoma treatment reducing simultaneously the glioblastoma reoccurrence.

Despite tremendous progress in molecular studies on cancer diagnostics and treatment [5,6], phyto-products extracted and isolated from medicinal plants and herbs, including Cannabis, are still a rich source of naturally occurring therapeutic compounds for experimental oncology [7,8,9]. Although the precise anti-cancer effects of cannabinoids are unknown as yet [10,11], advanced experimental studies could shed more light on cancer therapy, if the experiments are properly designed and conducted.

Accumulating experimental evidence indicates that some cannabinoids, including cannabidiol (a non-psychoactive naturally occurring cannabinoid [12], which is a weak G-protein-coupled receptor 55 (GPR55) and cannabinoid CB_1_ receptor antagonist [13], cannabinoid CB_2_ receptor inverse agonist [14], inhibitor of anandamide uptake, and a weak agonist at TRP vanilloid receptors [15]), possess anti-viability effects in various glioblastoma cell lines [16,17,18].

Experimental in vitro studies based on glioblastoma cell lines revealed that cannabidiol, AM1172, arvanil, olvanil, ACEA, WIN 55,212-2 mesylate and LY-2183240 produced the anti-viability effects in various glioblastoma and neuroblastoma cell lines in the MTT assays [19,20,21]. Moreover, in vitro experiments revealed that various cannabinoid receptor ligands (arvanil, olvanil, AM1172, ACEA, WIN 55,212-2 mesylate, LY-2138240) produced all types of interactions when combined with temozolomide in various neuroblastoma and glioblastoma cell lines [19,20,21]. Similarly, the anti-viability effects produced by the combinations of cannabidiol and temozolomide in two-drug mixture have been tested experimentally in in vitro studies; however, the combinations produced some contradictory results displaying either additive or synergistic interactions [11,16]. More specifically, the combinations of cannabidiol with temozolomide in three human glioblastoma cell lines (U-87MG, T98G and U251) in the MTT assay produced additive interactions, as determined with the checkerboard assay and the fractional inhibitory concentration [16]. In contrast, the combinations of cannabidiol with temozolomide in two human glioblastoma cell lines (U373-V and U373-M) in the MTT assay produced synergistic interactions, as determined with the combination index calculation [11]. Although both temozolomide and cannabidiol, when used separately, produced clear-cut anti-viability effects in various glioblastoma and neuroblastoma cell lines, their combinations may produce different interactions depending on the models used for evaluating interactions and calculating synergy score values.

Generally, one can distinguish five different reference models used when determining types of pharmacodynamic interactions between drugs: the zero interaction potency (ZIP), Bliss, Loewe, highest single agent (HAS), and Bliss–Loewe consensus models. The ZIP model quantifies how the shape and potency (IC_50_) of a single-drug dose–response curve change when a second drug is introduced and synergy is observed if the expected dose–response corresponding to the combination is more potent/effective than expected and if the single drugs did not mutually affect the potency of each other [22]. The Bliss independence model quantifies the combined effect of two drugs acting through independent mechanisms and calculates expected combined effects based on the individual drugs’ activities [23]. The Loewe model defines the combined effect of two drugs under the assumption that the drugs are mutually interchangeable, the drugs represent the same compound, and combining similar drugs is equal to giving more of a single drug, assuming a predictable drug response to increasing concentrations [24]. The HAS model quantifies the combined effect of two drugs by comparing the effect to the most potent individual drug acting alone, and synergy is defined as the excess over the maximum response achieved with a single drug [22]. The Bliss–Loewe consensus model is a novel synergy scoring model combining three reference models (Bliss, Loewe, and HSA) that quantifies the expected combination effect and then calculates a consensus distribution that is the maximum expected combination effect among the models at each drug combination dose pair [25]. Considering the existence of five different referential models used while determining drug–drug pharmacodynamic interactions, it was of importance to test the combination of cannabidiol and temozolomide in all the available models (ZIP, Bliss, Loewe, HAS and Bliss–Loewe) to select the best model, which could be used in future in in vitro studies.

The first aim of this study was to assess the interactions between cannabidiol and temozolomide in various glioblastoma and neuroblastoma cell lines with respect to the impact of these drugs on the anti-viability effects in the MTT assay so as to characterize the nature (types) of interactions between the drugs and to recommend some favorable combinations to further clinical conditions. The second goal of this study was to compare analytical methods used experimentally to detect the types of interactions between two drugs so as to select the most adequate and proper method for the evaluation of in vitro interactions in future. Recently, in the anti-cancer in vitro studies, several methods allowing characterization of interactions are applied, but only a few are worthy of recommendation [25,26,27]. One of these methods is a classic log-probit isobolographic analysis of interaction, considered as a gold-standard method in preclinical verification of interactions [26,28], and the second recommended method is the Bliss–Loewe consensus method related to the application of Synergy Finder 3.0 online software, allowing automatic assessment and visualization of interactions as a heat-map illustrating interactions for various concentrations of drugs used in various two-drug mixtures [25]. Although both analytical methods are used in experimental oncology [29,30], a direct comparison of obtained results with these methods has never been performed. This is the principal reason for applying both analytical methods to assess the interactions between cannabidiol and temozolomide in the MTT assay so as to display any possible differences among the methods when verifying the in vitro interactions. In this study, interactions between cannabidiol and temozolomide in six various neuroblastoma and glioblastoma (CHP-134, KELLY, SF295, U-87MG, T98G, C6) cell lines were analyzed. To exclude or confirm some cannabidiol-mediated apoptotic effects, Western blots displayed expression of various proteins, including p21, p18, cyclin D1, Bax, Bcl-2. Additionally, with Western blotting, the expression of cannabinoid CB_1_ receptor protein after treatment with cannabidiol was confirmed in the SF295 and U-87MG cell lines. The invasiveness and cell migration potential of the tested neuroblastoma and glioblastoma cells treated with cannabidiol, temozolomide and their combination were verified in the wound healing (scratch) assay.

## 2. Results

### 2.1. Determination of IC_50_ Values for Cannabidiol and Temozolomide in the MTT Assay

In the MTT assay, both cannabidiol and temozolomide (when used separately) exerted clear-cut anti-viability effects in all the tested neuroblastoma and glioblastoma cell lines. Based on the same original concentration–response anti-viability effect matrices for cannabidiol and temozolomide, as determined in the MTT assay, median inhibitory concentrations (IC_50_ values) were calculated in various tested cell lines according to the log-probit and Synergy Finder 3.0 methods. The experimentally determined IC_50_ values for cannabidiol ranged from 3.85 to 5.76 µg/mL according to Synergy Finder 3.0 and from 3.16 to 5.78 µg/mL according to the log-probit method (Table 1). Similarly, temozolomide produced definite anti-viability effects in the MTT assay, and its IC_50_ values ranged from 38.89 to 295.16 µg/mL according to Synergy Finder 3.0 and from 41.58 to 303.90 µg/mL according to the log-probit method (Table 1).

### 2.2. Concentration–Response Analysis with Synergy Finder 3.0

#### 2.2.1. Combination Analysis of Interactions in the CHP-134 Neuroblastoma Cell Line

**Figure 1:**
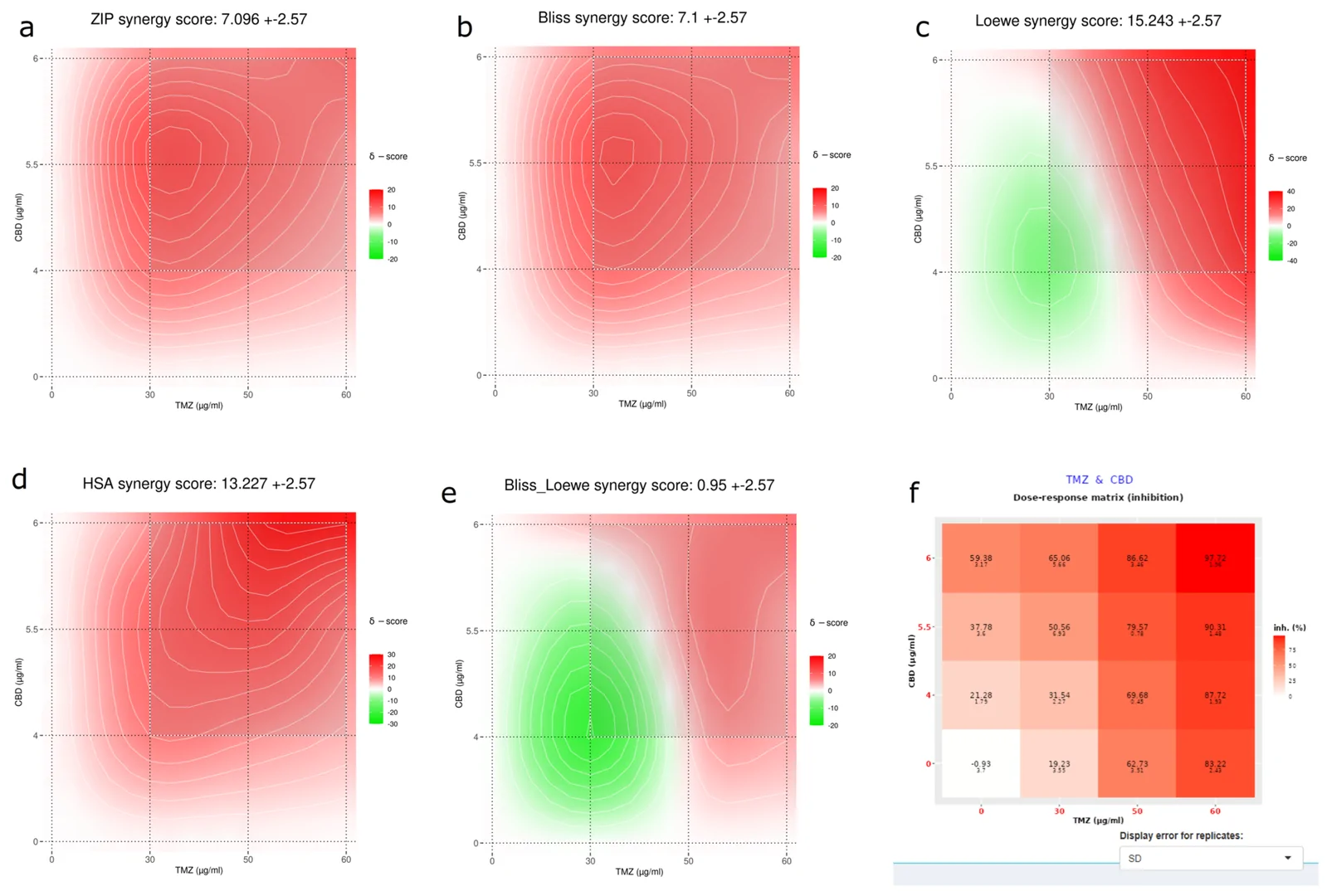
Graphical illustration of interactions between cannabidiol (CBD) and temozolomide (TMZ) in the ZIP (**a**), Bliss (**b**), Loewe (**c**), HAS (**d**), and Bliss–Loewe (**e**) models along with concentration–response viability effects of cannabidiol (CBD) and temozolomide (TMZ) in the CHP-134 neuroblastoma cell line in the MTT assay (**f**).

With Synergy Finder 3.0, it was possible to analyze a matrix comprising nine (3 × 3) variable-ratio concentrations for the combination of cannabidiol with temozolomide in the neuroblastoma CHP-134 cell line in the MTT assay. The automatic calculation of the synergy score in the ZIP model (7.10 ± 2.57) for this two-drug combination in the CHP-134 cell line revealed the existence of additive interactions between cannabidiol and temozolomide in this cell line (Figure 1a). An identical synergy score value (7.10 ± 2.57) was calculated automatically in the Bliss model, indicating the existence of additive interactions between cannabidiol and temozolomide in the CHP-134 cell line (Figure 1b). In the Loewe model, the synergy score value (15.24 ± 2.57) revealed the existence of synergistic interactions between cannabidiol and temozolomide in the CHP-134 cell line (Figure 1c). However, cannabidiol and temozolomide in the mixture, both at low concentrations, exerted antagonistic interactions (green area in the lower-left segments in Figure 1c), whereas at high concentrations of both drugs, the mixture produced the synergistic interactions in the MTT assay (red area in the upper-right segments in Figure 1c). In the HAS model, the synergy score value (13.23 ± 2.57) indicated the existence of synergistic interactions between cannabidiol and temozolomide in the CHP-134 cell line (Figure 1d). In contrast, in the Bliss–Loewe consensus model, the automatic calculation of the synergy score (0.95 ± 2.57) for this two-drug combination in the CHP-134 cell line revealed the existence of additive interactions between cannabidiol and temozolomide in this cell line (Figure 1e). More specifically, cannabidiol and temozolomide in the mixture at low concentrations exerted antagonistic interactions (green area in the lower-left segments in Figure 1e), whereas the two-drug mixtures at high concentrations produced the synergistic interactions in the MTT assay (red area in the upper-right segments in Figure 1e). A matrix of nine (3 × 3) various combinations of cannabidiol and temozolomide analyzed with Synergy Finder 3.0 along with their respective anti-viability effects, as estimated in the CHP-134 cell line in the MTT assay, is illustrated in Figure 1f.

#### 2.2.2. Combination Analysis of Interactions in the KELLY Neuroblastoma Cell Line

**Figure 2:**
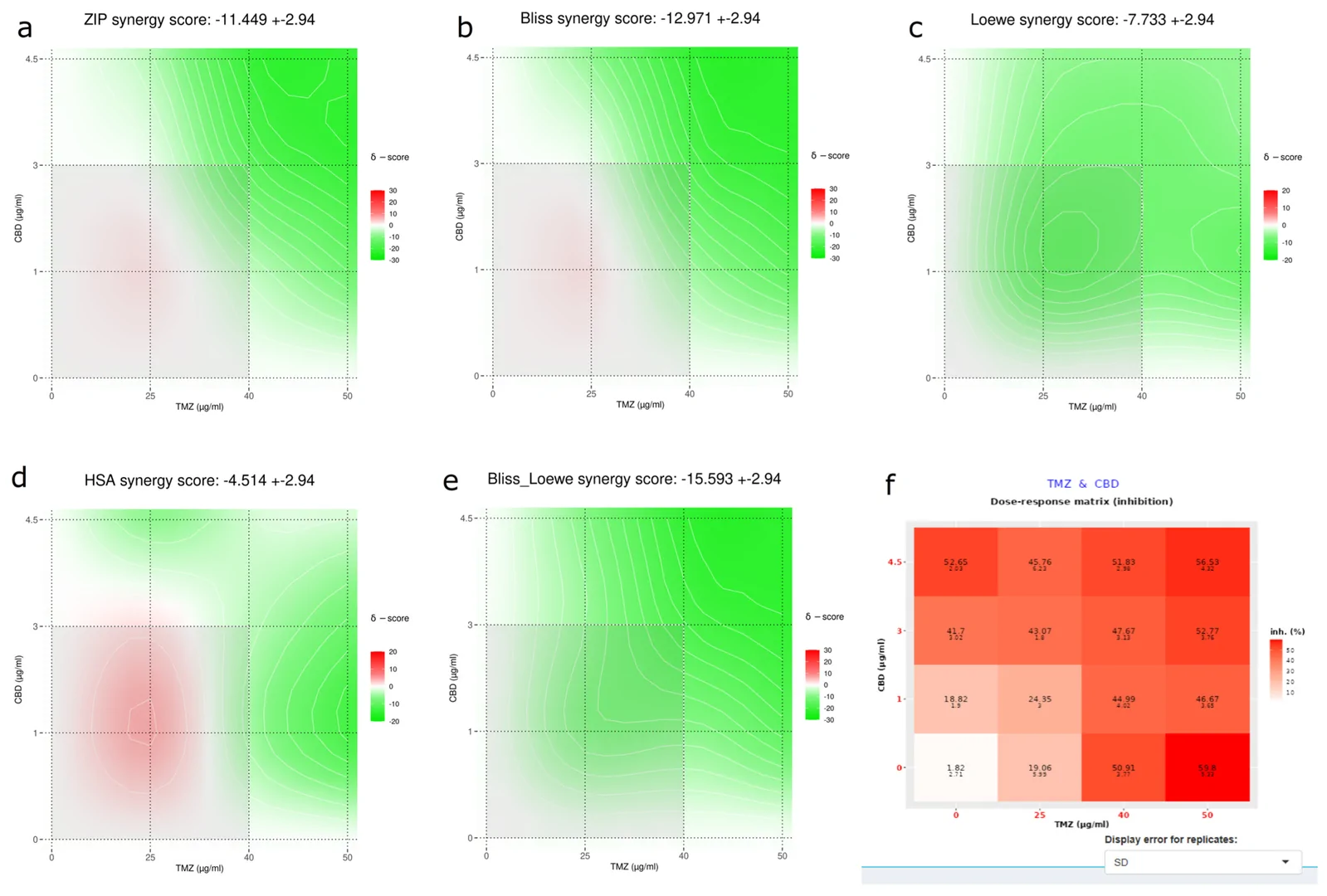
Graphical illustration of interactions between cannabidiol (CBD) and temozolomide (TMZ) in the ZIP (**a**), Bliss (**b**), Loewe (**c**), HAS (**d**), and Bliss–Loewe (**e**) models along with concentration–response viability effects of cannabidiol (CBD) and temozolomide (TMZ) in the KELLY neuroblastoma cell line in the MTT assay (**f**).

With Synergy Finder 3.0, it was possible to analyze a matrix consisting of nine various concentrations of two-drug mixtures for the combination of cannabidiol with temozolomide in the KELLY neuroblastoma cell line in the MTT assay. The automatic calculation of the synergy score in the ZIP model (−11.45 ± 2.94) for the combination of cannabidiol with temozolomide in the KELLY cell line revealed the existence of antagonistic interactions between both drugs in this cell line (Figure 2a). Similarly, the synergy score value (−12.97 ± 2.94) calculated automatically in the Bliss model revealed the existence of antagonistic interactions between cannabidiol and temozolomide in the KELLY cell line (Figure 2b). In the Loewe model, the synergy score value (−7.73 ± 2.94) revealed the existence of additive interactions between cannabidiol and temozolomide in the KELLY cell line (Figure 2c). In the HAS model, the synergy score value (−4.51 ± 2.94) indicated the existence of additive interactions between cannabidiol and temozolomide in the KELLY cell line (Figure 2d). However, cannabidiol and temozolomide in the mixtures at low concentrations exerted synergistic interactions (red area in the lower-left segments in Figure 2d), whereas the two-drug mixtures at high concentrations of both drugs produced antagonistic interactions in the MTT assay (green area in the upper-right segments in Figure 2d). In contrast, in the Bliss–Loewe consensus model, the automatic calculation of the synergy score (−15.59 ± 2.94) for this two-drug combination in the KELLY cell line revealed the existence of antagonistic interactions between cannabidiol and temozolomide in this cell line (Figure 2e). The strength of antagonistic interaction raised along with the increase in concentrations of the tested drugs in the MTT assay, becoming maximal at the highest tested concentrations of cannabidiol and temozolomide in the mixture (the most intensive green area in the upper-right segments in Figure 2e). A matrix of nine various combinations of cannabidiol and temozolomide analyzed with Synergy Finder 3.0 along with their respective anti-viability effects, as estimated in the KELLY cell line in the MTT assay, is illustrated in Figure 2f.

#### 2.2.3. Combination Analysis of Interactions in the SF295 Glioblastoma Cell Line

**Figure 3:**
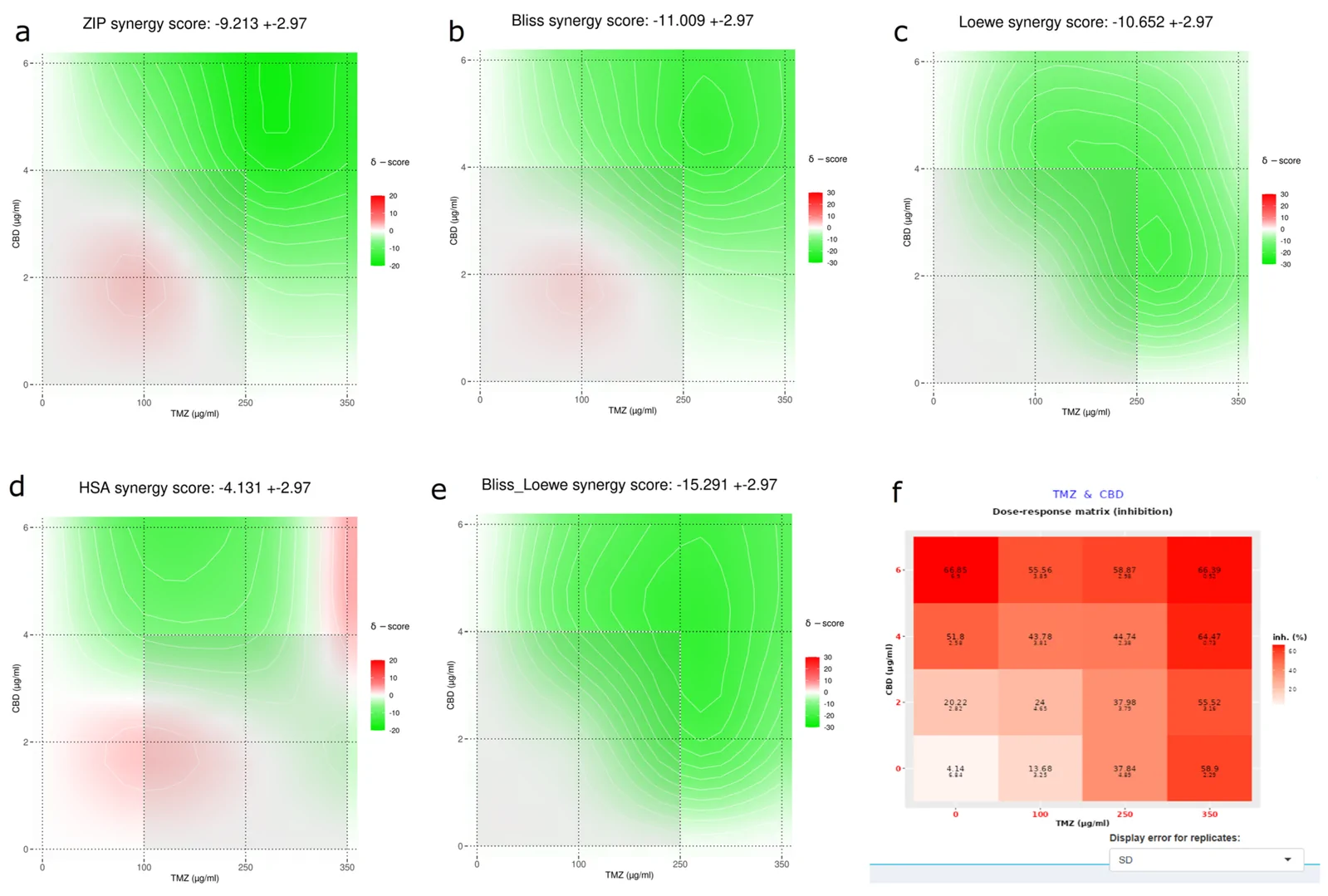
Graphical illustration of interactions between cannabidiol (CBD) and temozolomide (TMZ) in the ZIP (**a**), Bliss (**b**), Loewe (**c**), HAS (**d**), and Bliss–Loewe (**e**) models along with concentration–response viability effects of cannabidiol (CBD) and temozolomide (TMZ) in the SF295 glioblastoma cell line in the MTT assay (**f**).

A matrix of nine various concentrations of two-drug mixtures for the combination of cannabidiol with temozolomide in the SF295 glioblastoma cell line in the MTT assay was analyzed with Synergy Finder 3.0. The automatic calculation of the synergy score value in the ZIP model (−9.21 ± 2.97) for the combination of cannabidiol with temozolomide in the SF295 cell line revealed the existence of antagonistic and additive interactions between both drugs in this cell line (Figure 3a). However, cannabidiol and temozolomide in the mixtures at low concentrations exerted additive interactions (reddish and white areas in the lower-left segments in Figure 3a), whereas the two-drug mixtures at high concentrations of both drugs produced antagonistic interactions in the MTT assay (green area in the upper-right segments in Figure 3a). Similarly, the synergy score value (−11.01 ± 2.97) calculated automatically in the Bliss model revealed the existence of antagonistic and additive interactions between cannabidiol and temozolomide in the SF295 cell line (Figure 3b). However, cannabidiol and temozolomide in the mixtures at low concentrations exerted additive interactions (reddish and white areas in the lower-left segments in Figure 3b), whereas the two-drug mixtures at high concentrations of both drugs produced antagonistic interactions in the MTT assay (green area in the upper-right segments in Figure 3b). In the Loewe model, the synergy score value (−10.65 ± 2.97) revealed the existence of antagonistic interactions between cannabidiol and temozolomide in the SF295 cell line (Figure 3c). In the HAS model, the synergy score value (−4.13 ± 2.97) indicated the existence of additive interactions between cannabidiol and temozolomide in the SF295 cell line (Figure 3d). However, cannabidiol and temozolomide in the mixtures at low concentrations exerted additive interactions (reddish and white areas in the lower-left segments in Figure 3d), whereas the two-drug mixtures at high concentrations of both drugs produced antagonistic interactions in the MTT assay (green area in the upper-right segments in Figure 3d). In contrast, in the Bliss–Loewe model, the automatic calculation of the synergy score (−15.29 ± 2.97) for this two-drug combination in the SF295 cell line revealed the existence of antagonistic interactions between cannabidiol and temozolomide in this cell line (Figure 3e). The strength of antagonistic interaction raised along with the increase in concentrations of the tested drugs in the MTT assay, becoming maximal at the highest tested concentrations of cannabidiol and temozolomide in the mixture (the most intensive green area in the upper-right segments in Figure 3e). A matrix of nine various combinations of cannabidiol and temozolomide analyzed with Synergy Finder 3.0 along with their respective anti-viability effects, as estimated in the SF295 glioblastoma cell line in the MTT assay, is illustrated in Figure 3f.

#### 2.2.4. Combination Analysis of Interactions in the U-87MG Glioblastoma Cell Line

**Figure 4:**
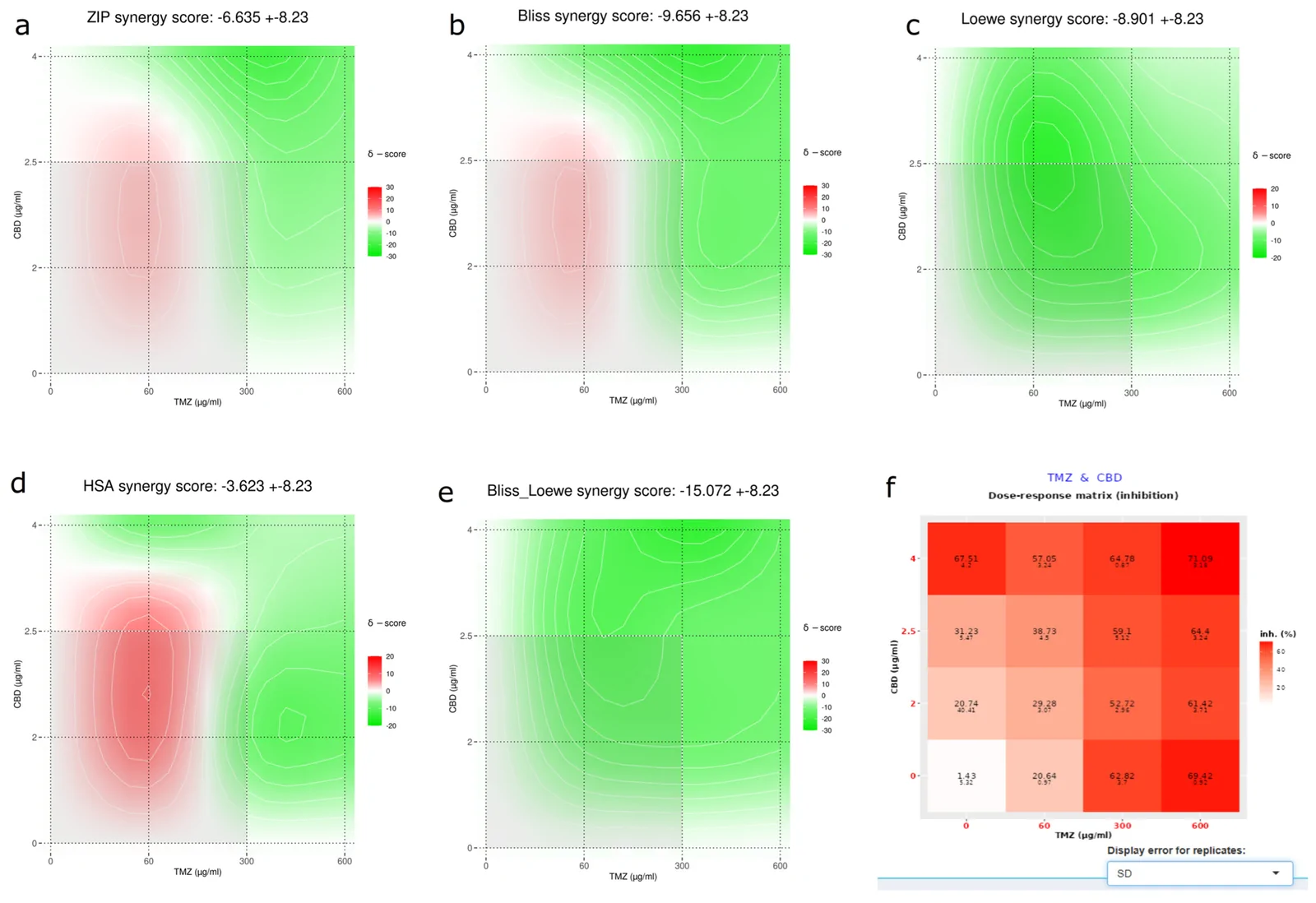
Graphical illustration of interactions between cannabidiol (CBD) and temozolomide (TMZ) in the ZIP (**a**), Bliss (**b**), Loewe (**c**), HAS (**d**), and Bliss–Loewe (**e**) models along with concentration–response viability effects of cannabidiol (CBD) and temozolomide (TMZ) in the U-87MG glioblastoma cell line in the MTT assay (**f**).

A matrix of nine various concentrations of two-drug mixtures for the combination of cannabidiol with temozolomide in the U-87MG glioblastoma cell line in the MTT assay was analyzed with Synergy Finder 3.0. The automatic calculation of the synergy score value in the ZIP model (−6.64 ± 8.23) for the combination of cannabidiol with temozolomide in the U-87MG cell line revealed the existence of additive interactions between both drugs in this cell line (Figure 4a). However, cannabidiol and temozolomide in the mixtures at low concentrations exerted additive interactions (red and white areas in the lower-left segments in Figure 4a), whereas the two-drug mixtures at high concentrations of both drugs produced antagonistic interactions in the MTT assay (green area in the upper-right segments in Figure 4a). Similarly, the synergy score value (−9.66 ± 8.23) calculated automatically in the Bliss model revealed the existence of antagonistic interactions between cannabidiol and temozolomide in the U-87MG cell line (Figure 4b). However, both drugs in the mixtures at low concentrations exerted additive interactions (red and white areas in the lower-left segments in Figure 4b), whereas the two-drug mixtures at high concentrations of both drugs produced antagonistic interactions in the MTT assay (green area in the upper-right segments in Figure 4b). In the Loewe model, the synergy score value (−8.90 ± 8.23) revealed the existence of additive interactions between cannabidiol and temozolomide in the U-87MG cell line (Figure 4c). In the HAS model, the synergy score value (−3.62 ± 8.23) indicated the existence of additive interactions between cannabidiol and temozolomide in the U-87MG cell line (Figure 4d). However, cannabidiol and temozolomide in the mixtures at low concentrations exerted additive interactions (red and white areas in the lower-left segments in Figure 4d), whereas the two-drug mixtures at high concentrations of both drugs produced antagonistic interactions in the MTT assay (green area in the upper-right segments in Figure 4d). In contrast, in the Bliss–Loewe model, the automatic calculation of synergy score value (−15.07 ± 8.23) for this two-drug combination in the U-87MG cell line revealed the existence of antagonistic interactions between cannabidiol and temozolomide in this cell line (Figure 4e). The strength of antagonistic interaction raised along with the increase in concentrations of the tested drugs in the MTT assay, becoming maximal at the highest tested concentrations of cannabidiol and temozolomide in the mixture (the most intensive green area in the upper-right segments in Figure 4e). A matrix of nine various combinations of cannabidiol and temozolomide analyzed with Synergy Finder 3.0 along with their respective anti-viability effects, as estimated in the U-87MG glioblastoma cell line in the MTT assay, is illustrated in Figure 4f.

#### 2.2.5. Combination Analysis of Interactions in the T98G Glioblastoma Cell Line

**Figure 5:**
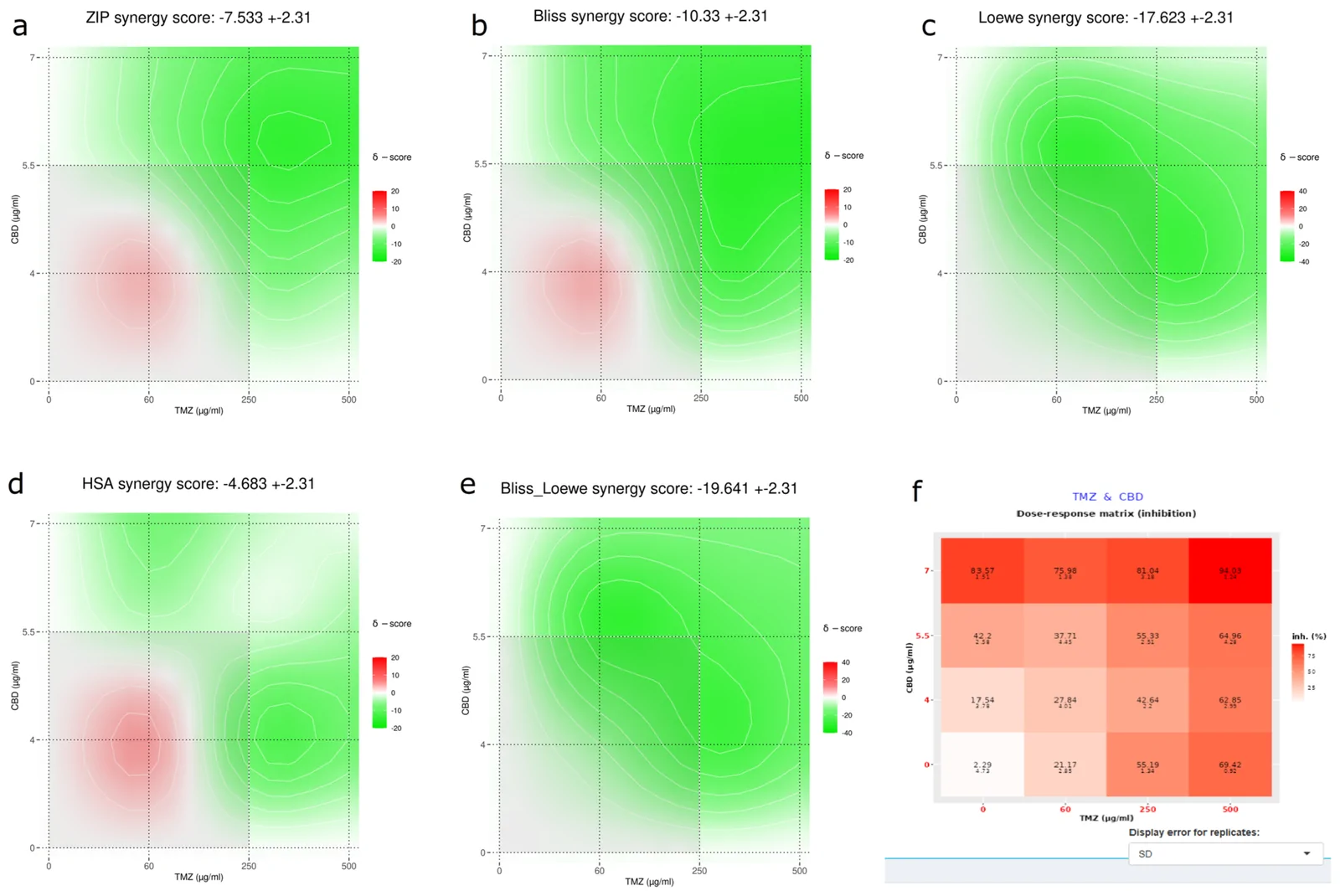
Graphical illustration of interactions between cannabidiol (CBD) and temozolomide (TMZ) in the ZIP (**a**), Bliss (**b**), Loewe (**c**), HAS (**d**), and Bliss–Loewe (**e**) models along with concentration–response viability effects of cannabidiol (CBD) and temozolomide (TMZ) in the T98G glioblastoma cell line in the MTT assay (**f**).

A matrix of nine various concentrations of two-drug mixtures for the combination of cannabidiol with temozolomide in the T98G glioblastoma cell line in the MTT assay was analyzed with Synergy Finder 3.0. The automatic calculation of the synergy score value in the ZIP model (−7.53 ± 2.97) for the combination of cannabidiol with temozolomide in the T98G cell line revealed the existence of additive interactions between both drugs in this cell line (Figure 5a). However, the mixtures of cannabidiol and temozolomide at low concentrations exerted additive interactions (reddish and white areas in the lower-left segments in Figure 5a), whereas the two-drug mixtures at high concentrations of both drugs produced antagonistic interactions in the MTT assay (green area in the upper-right segments in Figure 5a). Similarly, the synergy score value (−10.33 ± 2.31) calculated automatically in the Bliss model revealed the existence of antagonistic interactions between cannabidiol and temozolomide in the T98G cell line (Figure 5b). However, cannabidiol and temozolomide in the mixtures at low concentrations exerted additive interactions (reddish and white areas in the lower-left segments in Figure 5b), whereas the two-drug mixtures at high concentrations of both drugs produced antagonistic interactions in the MTT assay (green area in the upper-right segments in Figure 5b). In the Loewe model, the synergy score value (−17.62 ± 2.31) revealed the existence of antagonistic interactions between cannabidiol and temozolomide in the T98G cell line (Figure 5c). In the HAS model, the synergy score value (−4.68 ± 2.31) indicated the existence of additive interactions between cannabidiol and temozolomide in the T98G cell line (Figure 5d). However, cannabidiol and temozolomide in the mixtures at low concentrations exerted additive interactions (reddish and white areas in the lower-left segments in Figure 5d), whereas the two-drug mixtures at high concentrations of both drugs produced the antagonistic interactions in the MTT assay (green area in the upper-right segments in Figure 5d). In contrast, in the Bliss–Loewe model, the automatic calculation of the synergy score value (−19.64 ± 2.31) for this two-drug combination in the T98G cell line revealed the existence of antagonistic interactions between cannabidiol and temozolomide in this cell line (Figure 5e). The strength of antagonistic interaction raised along with the increase in concentrations of the tested drugs in the MTT assay, becoming maximal at the highest tested concentrations of cannabidiol and temozolomide in the mixture (the most intensive green area in the upper-right segments in Figure 5e). A matrix of nine various combinations of cannabidiol and temozolomide analyzed with Synergy Finder 3.0 along with their respective anti-viability effects, as estimated in the T98G glioblastoma cell line in the MTT assay, is illustrated in Figure 5f.

#### 2.2.6. Combination Analysis of Interactions in the C6 Glioma Cell Line

**Figure 6:**
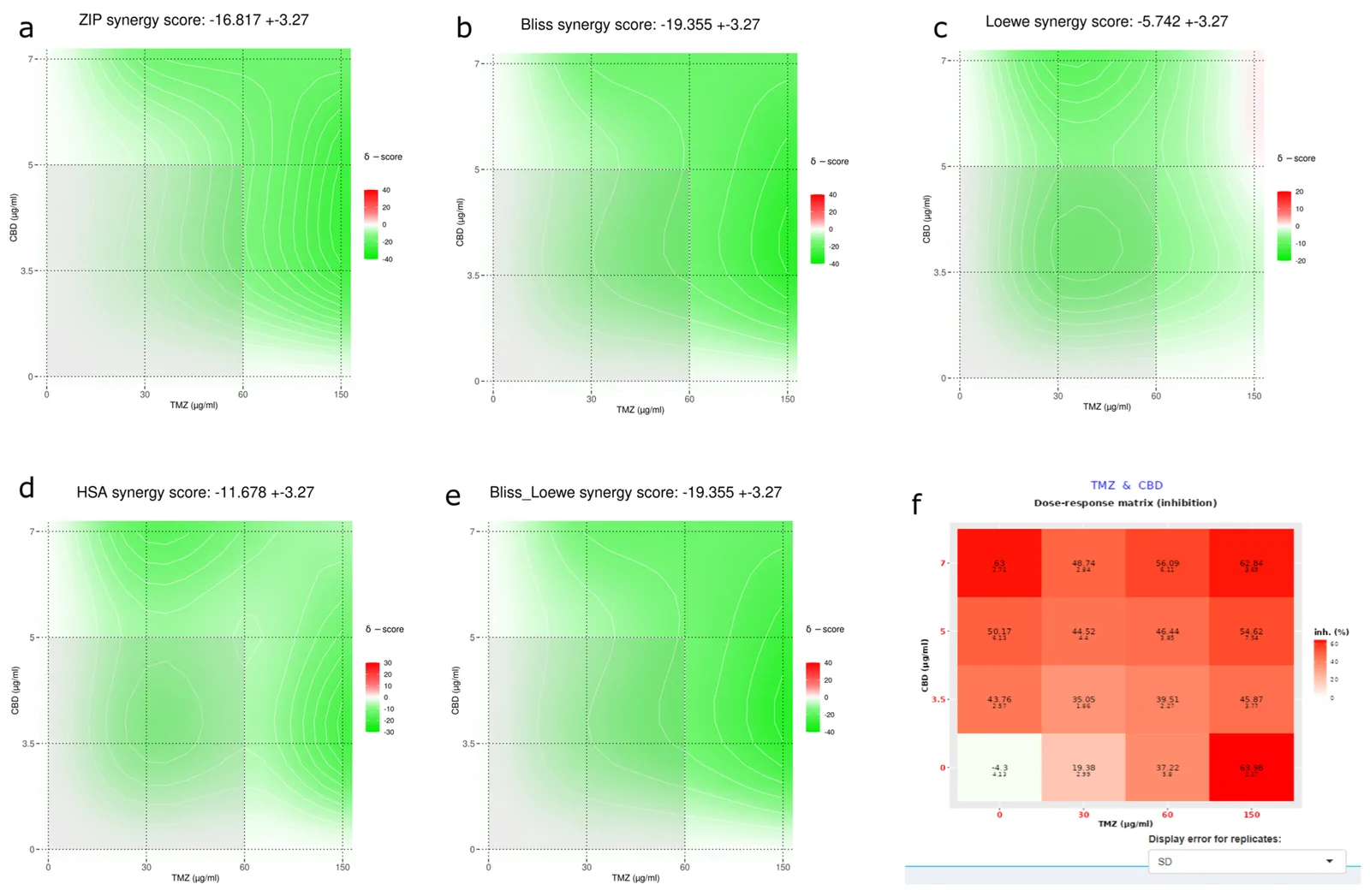
Graphical illustration of interactions between cannabidiol (CBD) and temozolomide (TMZ) in the ZIP (**a**), Bliss (**b**), Loewe (**c**), HAS (**d**), and Bliss–Loewe (**e**) models along with concentration–response viability effects of cannabidiol (CBD) and temozolomide (TMZ) in the C6 glioma cell line in the MTT assay (**f**).

A matrix of nine various concentrations of two-drug mixtures for the combination of cannabidiol with temozolomide in the C6 glioma cell line in the MTT assay was analyzed with Synergy Finder 3.0. The automatic calculation of the synergy score in the ZIP model (−16.82 ± 3.27) for the combination of cannabidiol with temozolomide in the C6 cell line revealed the existence of antagonistic interactions between both drugs in this cell line (Figure 6a). Similarly, the synergy score value (−19.36 ± 3.27) calculated automatically in the Bliss model revealed the existence of antagonistic interactions between cannabidiol and temozolomide in the C6 cell line (Figure 6b). In the Loewe model, the synergy score value (−5.74 ± 3.27) revealed the existence of additive interactions between cannabidiol and temozolomide in the C6 cell line (Figure 6c). In the HAS model, the synergy score value (−11.68 ± 3.27) indicated the existence of antagonistic interactions between cannabidiol and temozolomide in the C6 cell line (Figure 6d). Similarly, in the Bliss–Loewe model, the automatic calculation of the synergy score (−19.36 ± 3.27) for this two-drug combination in the C6 cell line revealed the existence of antagonistic interactions between cannabidiol and temozolomide in this cell line (Figure 6e). A matrix of nine various combinations of cannabidiol and temozolomide analyzed with Synergy Finder 3.0 along with their respective anti-viability effects, as estimated in the C6 glioma cell line in the MTT assay, is illustrated in Figure 6f.

### 2.3. Isobolographic Analysis of Interactions Between Cannabidiol and Temozolomide in the MTT Assay

The test for parallelism of log-probit concentration–response inhibitory effects of cannabidiol and temozolomide revealed that cannabidiol had its log-probit concentration–response inhibitory effect line collateral to that of temozolomide in two neuroblastoma (CHP-134 and KELLY) and two glioblastoma (SF295 and C6) cell lines and non-parallel in the remaining tested human glioblastoma (U-87MG and T98G) cell lines, respectively (Supplementary Materials, Figure S1a–f).

**Figure 7:**
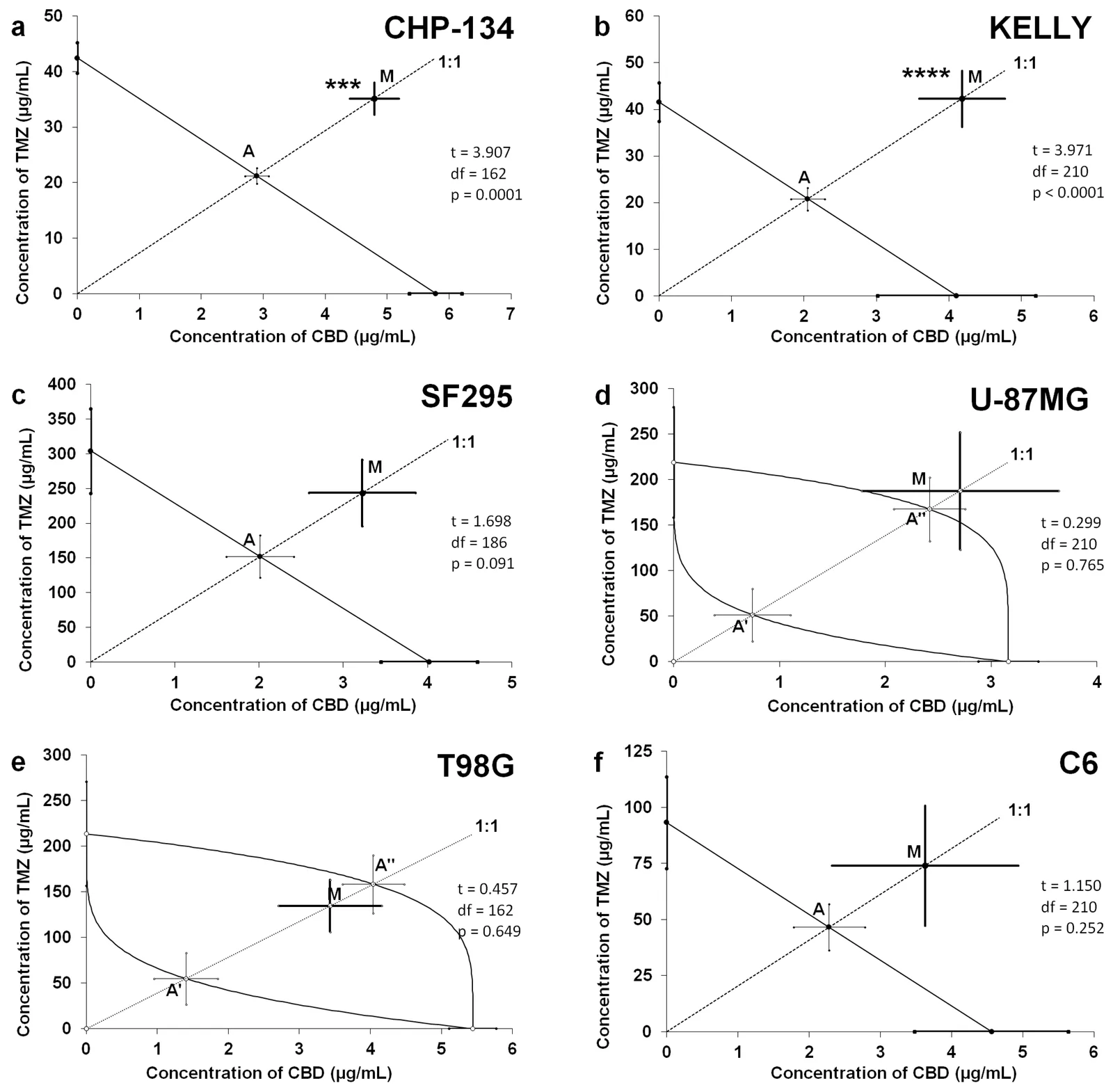
Interactions of cannabidiol (CBD) with temozolomide (TMZ) in the CHP-134 (**a**), KELLY (**b**), SF295 (**c**), U-87MG (**d**), T98G (**e**), and C6 (**f**) cell lines in the MTT assay. The IC_50_ (±SE) values for cannabidiol and temozolomide were plotted in the Cartesian system of coordinates. The theoretically additive IC_50add_ values were plotted graphically as the points A (for parallel isobolographic analysis) or A′, A″ (for non-parallel isobolographic analysis for lower (A′) and upper (A″) IC_50add,_ respectively), whereas the experimentally derived IC_50mix_ values are plotted as the point M on each multi-part graph. Statistical analysis of data was performed with the unpaired Student’s *t*-test. *** *p* < 0.001 and **** *p* < 0.0001 vs. the respective IC_50add_ values.

The isobolographic analysis of interactions performed with the log-probit method (based on the concentration–response anti-viability effects presented in the matrices for the respective cell lines) revealed that the combination of cannabidiol with temozolomide (at a fixed ratio of 1:1) exerted antagonistic interactions in two neuroblastoma (CHP-134 and KELLY) cell lines (Table 2; Figure 7a,b). More specifically, statistical analysis of data with Student’s *t*-test revealed that the experimentally derived IC_50mix_ value for the combination of cannabidiol with temozolomide in the neuroblastoma CHP-134 cell line significantly differed from the theoretically calculated IC_50add_ value, predicted to be additive, in the MTT assay (t_162_ = 3.907; *p* = 0.0001; Table 2). A graphical presentation of interaction for parallel concentration–response curves in the Cartesian system of coordinates illustrates that the experimentally derived IC_50mix_ value is placed significantly (*p* < 0.001) above the theoretically calculated IC_50add_ value (predicted to be additive) on the diagonal line representing the fixed ratio combination of 1:1 (Figure 7a).

Similarly, with Student’s *t*-test, the IC_50mix_ value for the combination of cannabidiol with temozolomide in the KELLY cell line considerably differed from the theoretically calculated IC_50add_ value in the MTT assay (t_210_ = 3.971; *p* < 0.0001; Table 2). In the Cartesian system of coordinates, the IC_50mix_ value for the mixture of cannabidiol with temozolomide in the MTT assay is placed significantly (*p* < 0.0001) above the theoretically calculated IC_50add_ value on the diagonal line representing the fixed ratio combination of 1:1 (Figure 7b).

In contrast, statistical analysis with Student’s *t*-test confirmed that the IC_50mix_ value for the combination of cannabidiol with temozolomide in the glioblastoma SF295 cell line did not differ significantly from the theoretically calculated IC_50add_ value in the MTT assay (t_186_ = 1.698; *p* = 0.091; Table 2). The isobologram reporting additive interaction with a tendency towards antagonism for the parallel concentration–response curve in the human glioblastoma SF295 cell line displays that the IC_50mix_ value for the mixture of cannabidiol with temozolomide in the MTT assay is placed above the theoretically calculated IC_50add_ value on the diagonal line representing the fixed ratio combination of 1:1 (Figure 7c).

Similarly, statistical analysis of data with Student’s *t*-test confirmed that the IC_50mix_ value for the combination of cannabidiol with temozolomide in the glioblastoma U-87MG cell line did not differ significantly from the theoretically calculated IC_50add_ value in the MTT assay (t_210_ = 0.299; *p* = 0.765; Table 3). However, the isobologram reporting interaction for a non-parallel concentration–response curve in the glioblastoma U-87MG cell line displays that the IC_50mix_ value for the mixture of cannabidiol with temozolomide in the MTT assay is placed above and simultaneously close to the theoretically calculated IC_50add_ value on the diagonal line representing the fixed ratio combination of 1:1 (Figure 7d). In such a situation, the additive interaction with a tendency towards antagonism can be ascribed to the combination of cannabidiol with temozolomide in this U-87MG cell line.

Likewise, with Student’s *t*-test, the IC_50mix_ value for the combination of cannabidiol with temozolomide in the glioblastoma T98G cell line did not differ from the theoretically calculated IC_50add_ value in the MTT assay (t_162_ = 0.457; *p* = 0.649; Table 3). In the Cartesian system of coordinates, the IC_50mix_ value for the mixture of cannabidiol with temozolomide in the MTT assay is placed within the area of additivity bounded by two lower and upper lines of additivity on the diagonal line representing the fixed ratio combination of 1:1 (Figure 7e).

The isobolographic analysis of interaction for a parallel concentration–response curve for the combination of cannabidiol with temozolomide in the last examined C6 cell line in the MTT assay revealed that the IC_50mix_ value for the mixture of both cannabidiol and temozolomide at a fixed-ratio of 1:1 did not differ significantly from the theoretically calculated IC_50add_ value in the MTT assay (t_210_ = 1.150; *p* = 0.252; Table 2). In the Cartesian system of coordinates, the IC_50mix_ value for the mixture of cannabidiol with temozolomide in the MTT assay is placed above the theoretically calculated IC_50add_ value indicating the additive interaction between drugs with a tendency towards antagonism (Figure 7f).

### 2.4. Molecular Docking of Cannabidiol, Temozolomide, and MTIC with the Cannabinoid CB_1_ Receptor

Molecular docking analysis was performed to investigate the predicted binding modes of cannabidiol, temozolomide, and its active metabolite MTIC: (5-(3-methyltriazen-1-yl)imidazole-4-carboxamide) within the cannabinoid CB_1_ receptor binding site. Two independent docking runs were conducted for each compound. The mean top-ranked predicted binding affinities were −8.15 kcal/mol for cannabidiol, −6.07 kcal/mol for temozolomide, and −5.50 kcal/mol for MTIC. Among the investigated compounds, cannabidiol demonstrated the most favorable predicted binding affinity, followed by temozolomide and MTIC. The docking scores obtained in the independent runs were highly consistent, with differences between the corresponding top-ranked poses of 0.004 kcal/mol for cannabidiol, 0.006 kcal/mol for temozolomide, and 0.006 kcal/mol for MTIC. The predicted binding affinities and the results of both docking runs are summarized in Table 4.

**Figure 8:**
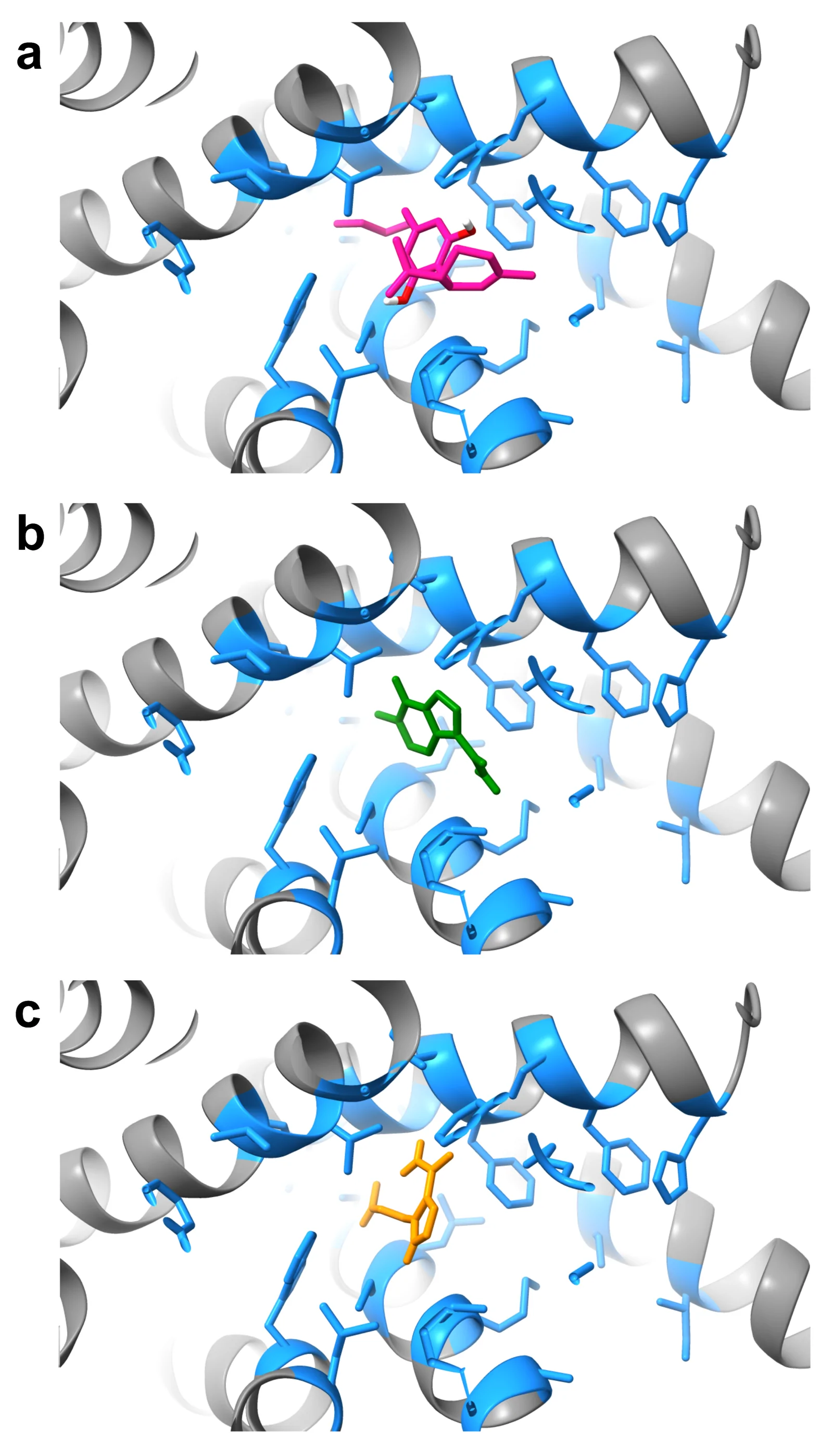
Predicted binding poses of cannabidiol, temozolomide, and MTIC within the cannabinoid CB_1_ receptor binding pocket. (**a**) Cannabidiol (pink), (**b**) temozolomide (green), and (**c**) MTIC (orange). Cannabinoid CB_1_ receptor is presented in blue and gray. The images illustrate the spatial positioning of the docked compounds within the receptor binding region. Each panel represents the best-ranked docking pose (mode 1) from the corresponding docking analysis.

The predicted interacting residues differed between the investigated ligands. For cannabidiol, the identified residues included Phe102, Ser383, Ser199, Gly166, Met103, Ile105, Leu387, and Phe268, while Ser390 and Cys386 were additionally identified in the second docking run. For temozolomide, the predicted interacting residues included Ser383, Gly166, Phe170, Val196, Leu387, and Cys386. The predicted MTIC binding site included Cys386, Gly166, Ser383, Phe170, Leu387, Ser390, and Trp356. The predicted binding orientations of the ligands within the cannabinoid CB_1_ receptor binding site are presented in Figure 8.

No potential hydrogen-bond interactions were detected for cannabidiol under the applied analysis conditions. For temozolomide, a potential hydrogen-bond interaction involving Ser383 was identified in both docking runs, with donor–acceptor distances of 3.262 Å and 3.230 Å, respectively. In the case of MTIC, potential hydrogen-bond interactions involving Cys386 and Gly166 were identified in both docking runs. For Cys386, donor–acceptor distances of 3.021 Å and 3.020 Å were obtained, whereas the corresponding distances for Gly166 were 3.171 Å and 3.158 Å in the first and second docking runs, respectively. These geometrical parameters were considered indicative of potential hydrogen-bonding interactions rather than definitive evidence of hydrogen-bond formation.

As a supplementary procedural check, the reference ligand ZDG (AM6538), present in the experimental cannabinoid CB_1_ receptor structure, was subjected to redocking after removal from the receptor binding site. The top-ranked redocked pose showed a predicted binding affinity of −12.866 kcal/mol. This analysis was performed to provide an additional check of the applied docking setup by comparing the predicted binding mode of the reference ligand with its experimentally determined position. The redocking analysis was not included in the comparative evaluation of cannabidiol, temozolomide, and MTIC and was not intended to establish experimental receptor–ligand binding or biological activity. The detailed docking results for cannabidiol, temozolomide, and MTIC are presented in Table 4 and Table S1, while the corresponding predicted binding modes are illustrated in Figure 8 and Figure S2.

### 2.5. Wound Healing Assay

**Figure 9:**
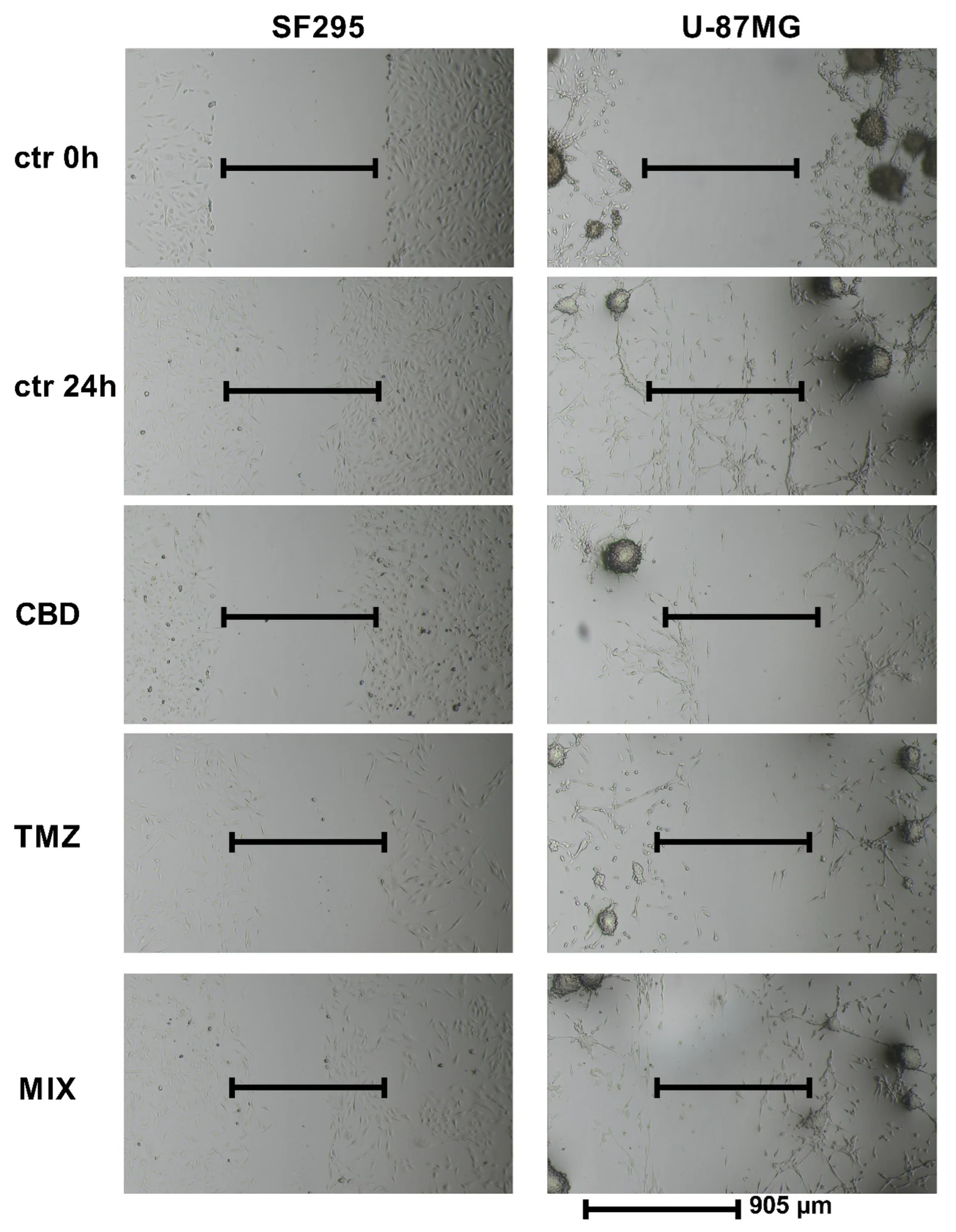
Images from a wound healing assay experiment on SF295 and U-87MG cell lines. Human glioblastoma cells (SF295 and U-87MG) were plated, wounded with a sterile pipette tip, and treated with cannabidiol (CBD), temozolomide (TMZ) and their combination (MIX), at the respective IC_50_ values, and then imaged 24 h after using an inverted microscope. Scale bar = 905 µm.

**Figure 10:**
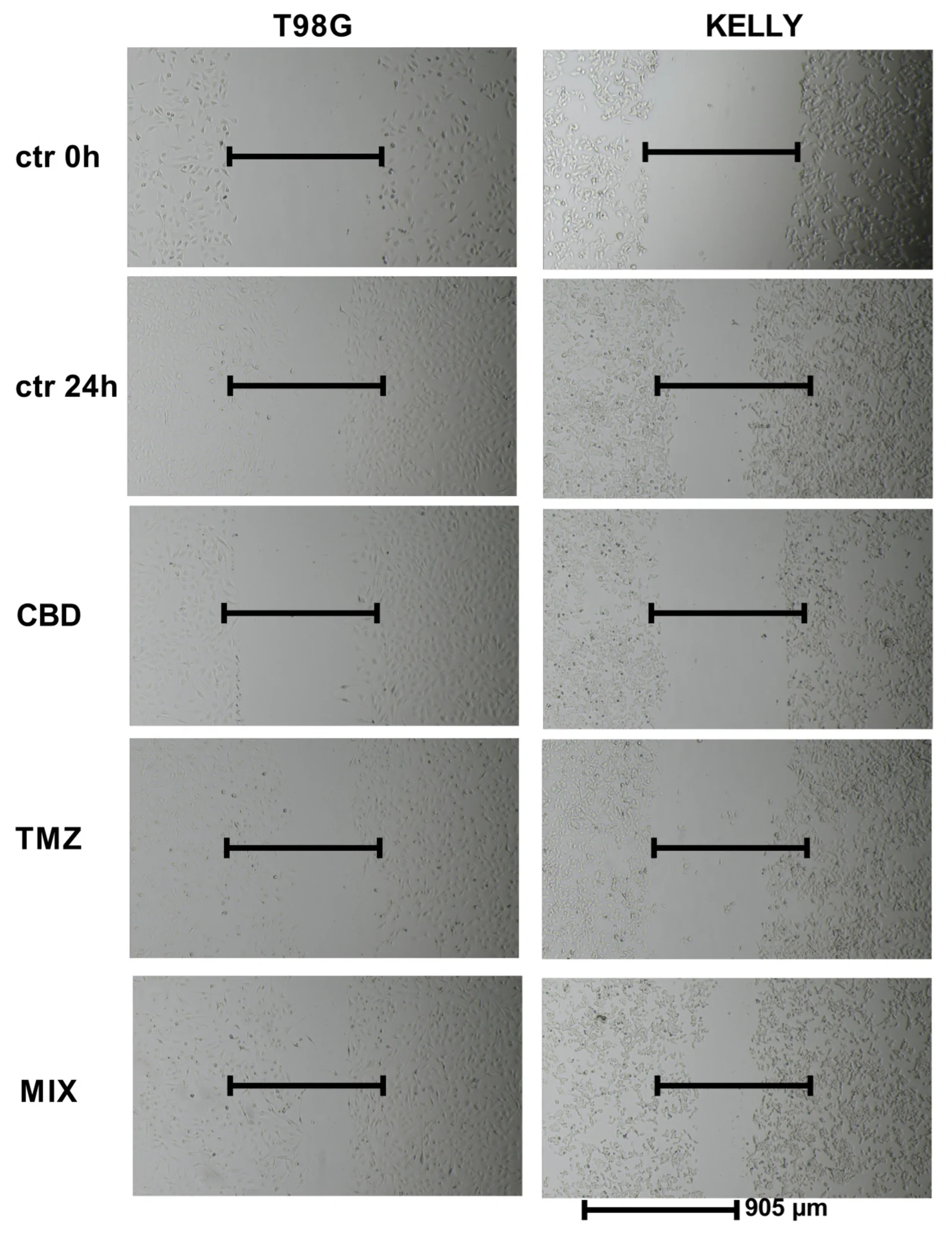
Images from a wound healing assay experiment on T98G and KELLY cell lines. Human glioblastoma (T98G) and neuroblastoma (KELLY) cells were plated, wounded with a sterile pipette tip, and treated with cannabidiol (CBD), temozolomide (TMZ) and their combination (MIX), at the respective IC_50_ values, and then imaged 24 h after using an inverted microscope. Scale bar = 905 µm.

**Figure 11:**
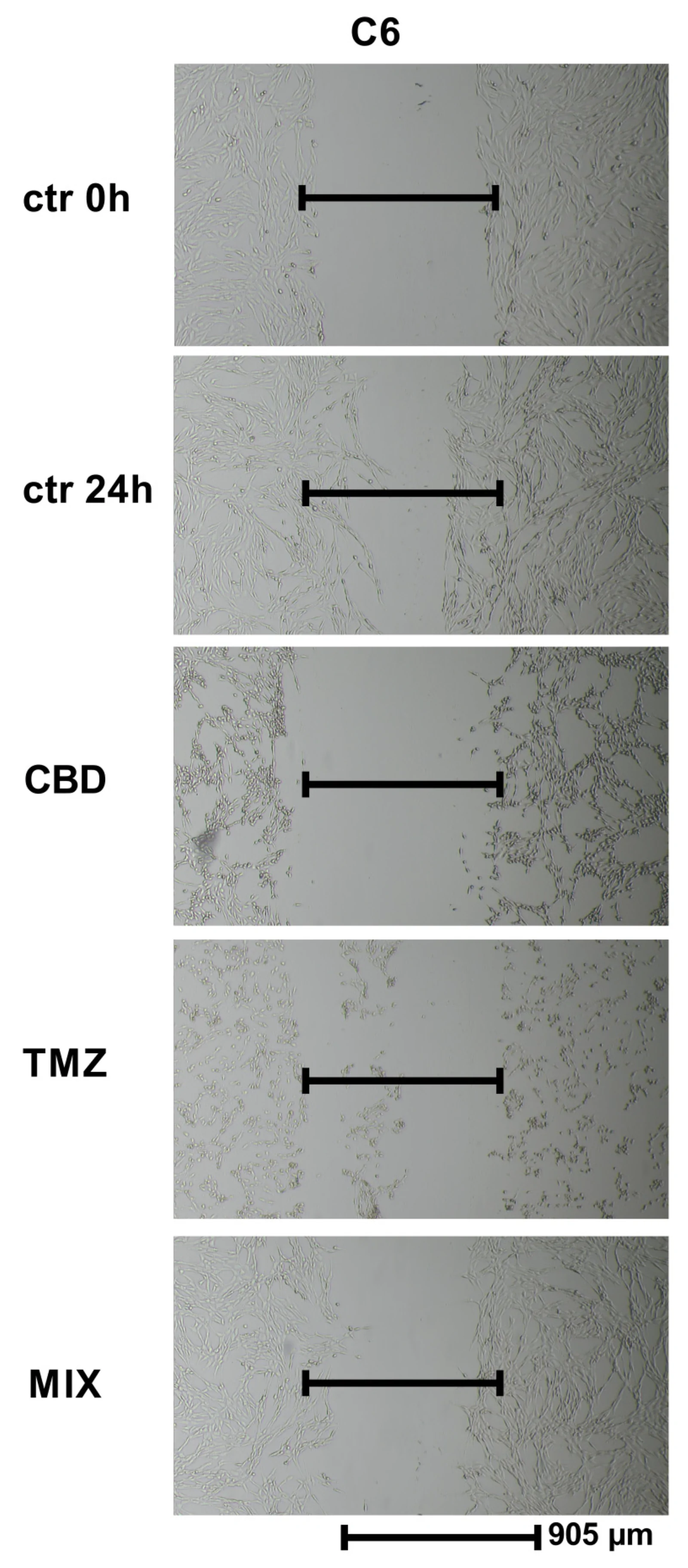
Images from a wound healing assay experiment on C6 cell line. Rat glioma (C6) cells were plated, wounded with a sterile pipette tip, and treated with cannabidiol (CBD), temozolomide (TMZ) and their combination (MIX), at the respective IC_50_ values, and then imaged 24 h after using an inverted microscope. Scale bar = 905 µm.

In the wound healing assay, it was found that cannabidiol reduced wound closure, suggesting an inhibitory effect on cell migratory capacity. However, cannabidiol at its IC_50_ concentration induced pronounced morphological changes indicative of cellular damage, which may have contributed to the observed reduction in wound closure (Figure 9, Figure 10 and Figure 11).

Temozolomide markedly inhibited cell proliferation but did not significantly affect cell migration. Interestingly, the combination of cannabidiol and temozolomide did not further inhibit wound closure; instead, it resulted in increased cell migration compared with cannabidiol treatment alone, with wound closure approaching the control condition. This finding is consistent with the antagonistic interaction observed between cannabidiol and temozolomide in the MTT (viability) assay.

### 2.6. Western Blotting Analysis

**Figure 12:**
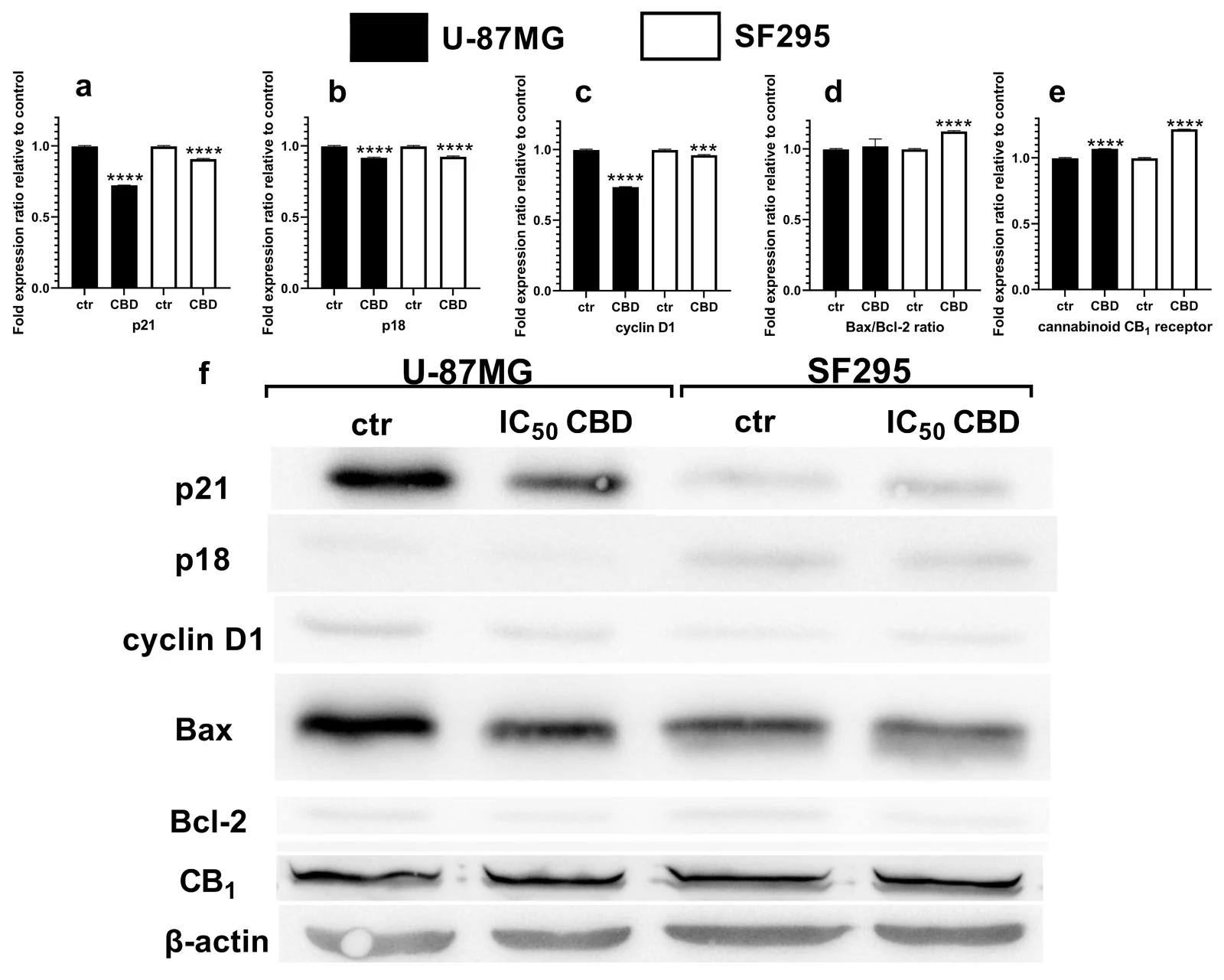
Western blots illustrating p21 (**a**), p18 (**b**), cyclin D1 (**c**), Bax and Bcl-2 (**d**), CB_1_ receptor (**e**), and β-actin (**f**) protein expression in glioblastoma (U-87MG and SF295) cell lines treated with either culture medium (ctr) or cannabidiol (at their respective IC_50_ concentrations). *** *p* < 0.001 and **** *p* < 0.0001 vs. the respective control (ctr) group.

With Western blotting, several apoptosis-regulating proteins were examined in the presence of cannabidiol. Two glioblastoma cell lines (U-87MG and SF295) with the lowest IC_50_ concentrations of cannabidiol were selected for these Western blotting experiments. Western blot analysis in the U-87MG cell line showed that cannabidiol at its IC_50_ concentration significantly reduced: (a) by ~30% p21 protein expression [t_4_ = 81.02; *p* < 0.0001]; (b) by ~10% p18 protein expression [t_4_ = 17.91; *p* < 0.0001]; (c) by ~25% cyclin D1 expression [t_4_ = 70.47; *p* < 0.0001] in the tested U-87MG cell line (Figure 12a–c). Similarly, Western blot analysis in the SF295 cell line showed that cannabidiol at its IC_50_ concentration significantly diminished: (a) by ~10% p21 protein expression [t_4_ = 20.84; *p* < 0.0001]; (b) by ~10% p18 protein expression [t_4_ = 16.23; *p* < 0.0001]; (c) by ~5% cyclin D1 expression [t_4_ = 8.781; *p* = 0.0009] in the tested SF295 cell line (Figure 12a–c). The concomitant reduction in p18, p21, and cyclin D1 expression suggests dysregulation of cell-cycle control, particularly at the G1/S transition. Although decreased cyclin D1 may impair cell-cycle progression, the reduction in the CDK inhibitors, p18 and p21, may indicate attenuation of cell-cycle inhibitory mechanisms.

In the U-87MG cell line, the ratio of Bax and Bcl-2 protein expression remained almost unchanged after treatment with cannabidiol [t_4_ = 0.6833; *p* = 0.5320], as compared to the control (Figure 12d). In contrast, Western blot analysis in the SF295 cell line revealed that the ratio of Bax and Bcl-2 protein expression significantly increased (by 10%) after treatment with cannabidiol as compared to the control [t_4_ = 26.48; *p* < 0.0001] (Figure 12d). A slight increase in the Bax/Bcl-2 ratio indicates only a modest shift toward a pro-apoptotic balance and, in the absence of additional apoptotic markers, does not provide unequivocal evidence of activation of the mitochondrial apoptotic pathway. Overall, these findings suggest that cannabidiol primarily affects cell-cycle regulatory mechanisms, with limited evidence for induction of apoptosis.

Western blot experiment confirmed also that treatment with cannabidiol (at its IC_50_ concentration) significantly increased the expression of cannabinoid CB_1_ receptors by 10% [t_4_ = 21.68; *p* < 0.0001] in the U-87MG and by 20% [t_4_ = 66.22; *p* < 0.0001] in the SF295 cell lines, respectively (Figure 12f). Treatment with cannabidiol increased cannabinoid CB_1_ receptor expression to approximately 1.2-fold compared with the control, suggesting a potential involvement of cannabinoid CB_1_-mediated signaling in the cellular response to the compound. However, the increase in cannabinoid CB_1_ receptor protein expression alone does not provide direct evidence of receptor activation.

## 3. Discussion

In this comparative study we have assessed the interactions between cannabidiol and temozolomide in the MTT assay by means of two methods: the log-probit isobolographic analysis [31] and Synergy Finder 3.0 software allowing the analysis of interactions for matrices of concentrations of two tested drugs (cannabidiol and temozolomide) based on five various pharmacodynamic models (ZIP, Bliss, Loewe, HAS, and the combined Bliss–Loewe consensus) [25]. Using the classic isobolographic analysis of interactions, we found that the combination of cannabidiol with temozolomide at the fixed ratio of 1:1 produced either antagonistic or additive interactions in the MTT assay depending on the cell lines used. More specifically, the combination of cannabidiol with temozolomide produced strong antagonistic interactions only in neuroblastoma CHP-134 and KELLY cell lines (*p* < 0.0001), whereas in glioblastoma SF295, U87MG, T98G and C6 cell lines, the combination of cannabidiol and temozolomide exerted additive interactions with a slight tendency towards antagonism.

One of the novel aspects of this study is the application of Synergy Finder 3.0 software—the freely available online platform allowing the automatic assessment of the types of interactions between the tested drugs (cannabidiol + temozolomide) along with estimation of proportions of the tested drugs offering the anti-cancer effects in the MTT assay. Using Synergy Finder 3.0, it is possible to select among various drug concentrations those offering favorable interactions in terms of the anti-viability effects in various in vitro glioblastoma cell lines [29]. With Synergy Finder 3.0, we have estimated automatically the types of interactions in five various pharmacodynamic models, including the ZIP, Bliss, Loewe, HAS and Bliss–Loewe consensus models. Unfortunately, the interactions of cannabidiol with temozolomide in the MTT assay considerably differed depending on the tested models (a brief summary of the reported interactions between cannabidiol and temozolomide in the respective models is presented in Table 5).

As shown in Table 5, the most restrictive pharmacodynamic model is the Bliss–Loewe consensus model reporting additive interactions in the neuroblastoma CHP-134 cell line and antagonistic interactions in all the remaining cell lines tested (KELLY, SF295, U-87MG, T98G and C6) in the MTT assay. Considering the fact that the Bliss–Loewe consensus model joins together three separate models (Bliss, Loewe and HAS) into one model, its application in this study becomes highly recommended as a model of choice when screening types of interactions in this in vitro study. By definition, the Bliss–Loewe consensus model combines the independence of the drugs tested (with respect to their mechanisms of action) with their mutual interchangeability (treated as adding more to the same drug) and evaluation of the anti-viability effects excessive over the maximal effects offered by one of the drugs used separately. This was the main reason to select this model as the preferential model in this study.

From a pharmacological viewpoint, any antagonistic interaction is unfavorable because higher concentrations of both drugs in the mixture are needed to produce the same therapeutic (anti-proliferative) effects. In this in vitro study, higher concentrations of two drugs were required to produce a 50% anti-viability effect, but these high concentrations might contribute to the increased risk of the appearance of adverse effects, negatively affecting the possible treatment in glioblastoma patients [32]. In other words, the two-drug combination reduced the anti-cancer effects, and higher concentrations of both drugs are required to maintain the desired anti-cancer effects at the same level (i.e., inhibition of the cell viability by 50%).

The antagonism between two drugs can be explained theoretically as an interaction leading to inactivation of molecular mechanisms of action of one drug by the second drug or competitive action of both drugs [33]. In molecular oncology, a competitive anti-cancer activity may occur if one drug blocks or arrests the cell cycle at an earlier stage (phase) required to trigger the molecular mechanism(s) of action of the second drug [34]. In such a case, the second drug cannot perform its anti-cancer action because the cells are not ready to be activated by the second drug. Thus, the first drug reduced the action of the second one, exerting finally the antagonistic interaction in the in vitro study. Notably, our findings reporting antagonistic interactions are against the theoretical expectations, since temozolomide is recommended as a drug of choice in glioblastoma patients with firmly confirmed efficacy in patients [35]. Similarly, cannabidiol when used alone produced favorable anti-cancer properties not only by inhibiting the cancer proliferation but also by contributing to improvement in the quality of life of the patients and thereby relieving the pain, inhibiting vomiting and nausea associated with chemotherapy, improving the quality of sleep, ameliorating appetite, exerting anti-depressant effects, etc. [36,37,38,39,40]. All these additional properties of cannabidiol make justified the choice of cannabidiol as a supportive drug in patients with glioblastoma. In addition, cannabidiol and other cannabinoids (including, THC and nabiximols) are naturally occurring phytocannabinoids, which are recommended in few clinical trials as adjunct and adjuvant treatment in glioblastoma patients (see: https://clinicaltrials.gov—NCT05629702; NCT03607643; NCT03529448). Theoretically, the anti-proliferative effects of both temozolomide and cannabidiol should mutually potentiate in terms of inhibiting cell viability due to various molecular mechanisms of action. Unfortunately, our experimental verification of interactions between cannabidiol and temozolomide in the MTT assay testifies against their combination in further therapy, and a special warning is advised not to combine cannabidiol with temozolomide in the treatment of glioblastoma and neuroblastoma patients.

The molecular docking results provide additional structural information concerning the potential interaction of the investigated compounds with the cannabinoid CB_1_ receptor; however, they do not establish a direct mechanistic relationship between the predicted ligand–receptor interactions and the observed pharmacodynamic antagonism. As illustrated in Figure 8, cannabidiol exhibited the most favorable predicted binding affinity among the investigated compounds, whereas temozolomide showed a less favorable predicted affinity. More specifically, the molecular docking analysis elucidated an unpredicted possibility of competitive binding of temozolomide and its active component MTIC to the cannabinoid CB_1_ receptors. Both temozolomide and MTIC have structural conformational ability to interfere with cannabinoid CB_1_ receptors. Theoretically, temozolomide and MTIC, by interfering with the active sites of the cannabinoid CB_1_ receptors, may disturb cannabidiol’s binding to the cannabinoid CB_1_ receptors, remaining neutral on these cannabinoid CB_1_ receptors. In such a situation, the receptor affinity of cannabidiol in the presence of temozolomide might be drastically reduced, and thus, cannabidiol was unable to produce its full anti-viability effects in the MTT assay, leading finally to antagonistic interactions in various neuroblastoma and glioblastoma cells. Although this hypothetical interpretation was not verified experimentally, it can readily explain the observed antagonistic interactions between drugs. The competitive presence of temozolomide and MTIC in specific target places (receptor active sites), where cannabidiol binds to and exerts its cannabinoid CB_1_ receptor-mediated effects, should be considered as a background of antagonistic interactions. Of note, the mutual presence of temozolomide and cannabidiol (when used in mixture) within the binding sites of cannabinoid CB_1_ receptors may evoke competitive removal of cannabidiol from its active places, and thus, the anti-viability effects of cannabidiol are suppressed, leading to antagonistic interactions, as observed in the MTT assay. Nevertheless, docking scores alone cannot be used to predict the biological outcome of a drug–drug combination, particularly when the compounds may act through different molecular targets and cellular pathways. The predicted binding of cannabidiol to cannabinoid CB_1_ receptors may therefore be considered a possible component of its pharmacological activity, but it does not provide sufficient evidence to conclude that cannabinoid CB_1_ receptor binding directly accounts for the antagonistic interaction observed between cannabidiol and temozolomide.

Notably, temozolomide and MTIC were both included in the docking analysis because of their direct pharmacological relationship. Temozolomide is an imidazotetrazine prodrug that undergoes spontaneous, pH-dependent conversion into the active metabolite MTIC under physiological conditions [41]. MTIC subsequently decomposes to generate a reactive methyldiazonium species responsible for DNA methylation, including methylation at the O6 position of guanine, which is considered particularly relevant to temozolomide-induced cytotoxicity [42,43]. Therefore, comparing the predicted interactions of temozolomide and MTIC with cannabinoid CB_1_ receptor was intended to explore whether the parent compound (temozolomide) and its active metabolite (MTIC) exhibited distinct potential binding modes within the receptor binding site. In the present study, temozolomide and MTIC demonstrated different predicted binding affinities and partially distinct predicted interacting residues. These observations may indicate differences in their predicted receptor-associated interaction profiles; however, they should be interpreted cautiously, as the principal cytotoxic activity of temozolomide/MTIC is associated with DNA alkylation and DNA damage rather than established direct binding to cannabinoid CB_1_ receptors. At present, there is no experimental proof confirming that temozolomide and MTIC affect directly the cannabinoid CB_1_ receptors, and further experimental studies would be required to confirm the hypothesis that temozolomide and/or its active metabolite (MTIC) directly modulate cannabinoid CB_1_ receptor-mediated signaling.

Unfortunately, in this study, none of the tested interactions between cannabidiol and temozolomide in various neuroblastoma and glioblastoma cell lines are beneficial and worthy of recommendation, except for those reported for the CHP-134 cell line in the Bliss–Loewe consensus model. In contrast, the classic isobolographic analysis of interactions revealed that both neuroblastoma cell lines (CHP-134 and KELLY) exerted antagonistic interactions in the MTT assay, whereas all the tested glioblastoma cell lines were additive. Of note, we used the same original concentration–response anti-viability effects matrices in both methods, the classic isobolographic analysis and Synergy Finder 3.0; however, the obtained results are divergent and considerably differed, making the methods incomparable. As reported earlier, the same two-drug combination could exert various types of interactions in various cell lines, confirming cell line-selectivity in the anti-viability effects [19,20,21], which indirectly suggested and justified the application of drugs in proportions offering the best anti-cancer effects for individual patients as a personalized oncologic treatment (POT) [44]. However, in this study we have displayed that the same two-drug combination tested on the same cell line in the same experimental conditions exerted different interactions depending on the model applied for the assessment of interactions.

It is important to note that IC_50_ concentrations of temozolomide and cannabidiol used in this study reached maximally 303.90 µg/mL in the SF295 cell line (for temozolomide), and 5.78 µg/mL in the CHP-134 cell line (for cannabidiol), which are equivalent to 1.56 mM (for temozolomide) and 18.38 µM (for cannabidiol), respectively. These concentrations are generally far higher than the achievable exposure levels in plasma and brain tissue in humans. The concentrations of drugs used in in vitro studies cannot easily be transferred to clinical conditions. Of note, such high concentrations of cannabidiol and temozolomide have never been achieved in clinical conditions. In such a situation the antagonistic interactions observed in in vitro studies would not occur in clinical settings. However, some molecular mechanisms observed in in vitro studies can be observed in clinical conditions despite a lack of direct translation of the tested doses/concentrations.

The main obstacle when analyzing the results with both methods was ascribed to various proportions of two drugs used in combination. The Synergy Finder 3.0 web-application software required several concentrations of every drug tested in combination, making finally a matrix of the tested two-drug concentrations (i.e., each concentration of the first drug was combined with each concentration of the second drug), resulting finally in several combinations of two drugs at constant concentrations, which can be readily illustrated in the Cartesian plot system of coordinates. This was the main reason to test several combinations in fixed concentrations so as to create a heat-map of the anti-viability effects in the MTT assay due to Synergy Finder 3.0. In contrast, in the classic isobolographic analysis of interaction, only a fixed-ratio of 1:1 was tested, for which drugs were used in equi-effective concentrations [31]. Additionally, the main limitations in this study are related to the in vitro experiments on cell lines without in vivo verification on animals or organoids/organs-on-chips. Generally, in vitro experiments on cell lines assess the anti-viability effects of the tested drugs without considering their distribution in living organisms. Concentrations applied in this in vitro study may never be reached in animals because, very often, concentrations suppressing cell proliferation in in vitro conditions are too high, producing some toxic effects by themselves when used in animals. On the other hand, none of the pharmacokinetic parameters of the tested drugs related to the liberation, absorption, distribution, metabolism and elimination (LADME) of the drugs, which finally affect concentrations of the drugs in the site of action, were tested in this in vitro study.

In contrast, although the log-probit method is scientifically proper, it allowed assessment of interaction only for the fixed-ratio combinations of both drugs used in equi-effective concentrations/doses [45]. The log-probit method needs more information before testing experimentally the two-drug mixtures, allowing for the proper calculations, whereas Synergy Finder 3.0 did not require previous presumptions and is based on concentrations of drugs that can be directly tested experimentally in in vitro conditions. Considering the results obtained from both methods, the combination of cannabidiol with temozolomide in the MTT assay should not be recommended clinically because of evident antagonism between drugs. The anti-proliferative effects underwent mutual inhibition, and thus, higher concentrations of both drugs are necessary to produce the desired 50% anti-viability effects.

Of note, the direct comparison of both methods clearly indicates that the log-probit isobolographic analysis required statistical analysis of data for comparing the theoretically calculated and predicted to be additive IC_50add_ values with the experimentally determined IC_50mix_ values. After statistical verification, the observed significant difference between these values indicates either synergistic or antagonistic interactions, depending on the IC_50mix_/IC_50add_ ratio, as documented earlier [31]. However, the lack of any significance confirms the additivity between the tested drugs. On the contrary, statistical analysis of data in Synergy Finder 3.0 has never been performed, and types of interactions are ascribed in relation to synergy score values calculated by this web-application. Synergy, antagonism and additivity are usually ascribed to the specific types of interactions based on specific fixed and predefined range values. More specifically, synergy score values lower than −10 (minus 10) are indicative of antagonism; values ranging from −10 (minus 10) to 10 are indicative of additive interactions, and values higher than 10 indicate synergistic interactions [25]. In our opinion, statistical analysis of data in the log-probit isobolographic analysis has greater power than the lack of statistics in Synergy Finder 3.0 based only on commonly accepted cutoff values, as a reference for synergy, antagonism and additivity. On the other hand, the Synergy Finder 3.0 seems to be more intuitive software, which does not require any additional statistical knowledge or special preparation for users who apply this online application. Minimal or no knowledge about statistical methods used during classification of interactions allowed unexperienced researchers and experimenters to apply this Synergy Finder 3.0 software without any presumptions required when analyzing interactions with log-probit isobolographic analysis. In addition, the Synergy Finder 3.0 platform is supported by artificial intelligence (AI) algorithms that boost accuracy and precision in detecting interactions without any assumptions before experimentally confirming the interactions.

In this study, we have also tested some cell migration properties of the drugs (cannabidiol and temozolomide) used either separately or in combination in the wound healing (scratch) assay. Progress in cell migration was assessed after 24 h of incubation with the respective drugs either alone or in combination. The results confirmed that the drugs in combination (i.e., cannabidiol and temozolomide) mutually inhibit their proper effects contributing to the enhanced cellular migration. In this wound healing assay we have confirmed the antagonistic effects evoked by the mixture of both cannabidiol and temozolomide in various cell lines, which may additionally disqualify the combination from further clinical recommendation.

## 4. Materials and Methods

### 4.1. Cell Cultures

In this in vitro study, 6 various cell lines—two human neuroblastoma (CHP-134 (RRID:CVCL_1124) and KELLY (RRID:CVCL_2092)—from the European Collection of Cell Cultures—ECACC, UK Health Security Agency, Salisbury, UK); three human glioblastoma (SF295 (RRID:CVCL_1690), U-87MG (RRID:CVCL_0022) and T98G (RRID:CVCL_0556)—from the American Type Culture Collection—ATCC, Manassas, VA, USA); and one rat malignant glioma (C6 (RRID:CVCL_0194) from ATCC, Manassas, VA, USA)—were used, as described earlier [19,20,21]. The growth conditions of the neoplastic culture were described in detail in the safety data sheet for each cell line (supported by ATCC and ECACC), as described earlier [19,20,21].

### 4.2. Drugs

Cannabidiol (Tocris, Bristol, UK) was dissolved in ethanol (at a maximal concentration of 0.1% *v*), whereas temozolomide (Sigma-Aldrich, St. Louis, MO, USA) was dissolved in sterile water for injection. Because ethanol in high concentrations is a potent neurotoxic agent [46], we used ethanol in this study at safe and low concentrations, which had no effect on cell growth and cell proliferation. For the two-drug mixtures, the control (untreated) cells were exposed to both solvents in the appropriate concentrations in all experiments.

### 4.3. MTT Assay

The impact of cannabidiol and temozolomide, when administered alone and in combination, on cell viability was assessed in the MTT assay, as described earlier [19,20]. All the tested cell lines—CHP-134, KELLY, SF295, U-87MG, T98G, and C6—were placed on 96-well plates (NEST Biotechnology, Wuxi, China; Catalog #:701001). After incubating the cell lines for 24 h, the culture medium was removed, and cells were exposed to serial dilutions of cannabidiol and temozolomide in fresh culture medium. Cell viability was assessed after 72 h by means of the MTT method in which the yellow tetrazolium salt (MTT; Sigma-Aldrich, St. Louis, MO, USA; Catalog #:M2128) is metabolized by viable cells to purple formazan crystals. Cells were incubated for 3 h with MTT solution (5 mg/mL). Formazan crystals were solubilized overnight in sodium dodecyl sulfate (SDS; Bio-Rad, Hercules, CA, USA; Catalog #:1610302) buffer (10% SDS in 0.01 N HCl), and the product was determined spectrophotometrically by measuring absorbance at 570 nm wavelength using a microplate spectrophotometer (Ledetect 96, Labexim Products, Lengau, Austria) [19,20,21]. The combinations of cannabidiol + temozolomide were tested in the CHP-134, KELLY, SF295, U-87MG, T98G, C6 cell lines, from which the experimentally derived IC_50mix_ values for the two-drug mixtures were determined in the MTT assay. Each experiment was performed in triplicate.

### 4.4. Analysis of Interactions by Synergy Finder 3.0

The synergy score values of two-drug combinations at different concentrations were calculated using Synergy Finder 3.0 software (https://synergyfinder.aittokallio.group/ accessed on 15 October 2026). The data for percent viability from the MTT assay by cannabidiol and temozolomide in multiple combinations were added in Synergy Finder 3.0 freely available web-application according to the user guide provided online. According to the recommendations presented in the user guide, a novel synergy scoring method (called Bliss/Loewe consensus) that combines multiple synergy reference models (Bliss, Loewe, and highest single agent) was selected, offering the best option for the detection of two-drug interactions. Subsequently, synergy score values for the combinations of cannabidiol with temozolomide in various cell lines were calculated. According to the Synergy Finder 3.0 user guide, synergy score values allowed the classification of interactions as follows: (a) if synergy score values are less than −10: the two-drug interaction is antagonistic; (b) if synergy score values range from −10 to 10: the interaction is likely to be additive; (c) if synergy score values are larger than 10: the two-drug interaction is likely to be synergistic.

### 4.5. Log-Probit Isobolographic Analysis

Interactions of cannabidiol with temozolomide in the tested cell lines were classified isobolographically in the MTT assay. The parallelism of the concentration–response lines for cannabidiol and temozolomide was verified in all the tested cell lines, as previously described [19,20,26]. From the already published IC_50_ values of cannabidiol and temozolomide (when administered alone in the MTT assay: Table 1), the median additive inhibitory concentrations for the mixtures (IC_50add_) (at a fixed ratio of 1:1) of cannabidiol + temozolomide were calculated, as described previously [20]. Next, the experimentally determined IC_50mix_ values for the combination of cannabidiol + temozolomide at the fixed ratio of 1:1 were calculated in approximation based on the log-probit method, as reported earlier [19]. To confirm the nature of interactions, a statistical comparison of the IC_50mix_ values with their respective IC_50add_ counterparts was performed as recommended elsewhere [26].

### 4.6. Molecular Docking Simulations

Molecular docking simulations were performed to investigate the potential binding modes and interactions of cannabidiol (CBD), temozolomide (TMZ), and its metabolite 5-(3-methyltriazen-1-yl)imidazole-4-carboxamide (MTIC) with the cannabinoid receptor type 1 (CB1). The crystal structure of the human CB_1_ receptor was obtained from the Protein Data Bank (PDB ID: 5TGZ) [47,48]. The receptor structure was prepared using UCSF ChimeraX (version 1.12) [49]. The protein structure was inspected, and the receptor was prepared for docking by removing unnecessary structural components and generating the receptor structure required for subsequent docking calculations.

The three-dimensional structures of CBD, TMZ, and MTIC were obtained in the structure data file (SDF) format and prepared for docking using Meeko (version 0.8.0) [50]. The prepared ligands were converted into the PDBQT format, including the assignment of atom types and partial charges required by AutoDock Vina [51]. The prepared CB1 receptor was also converted into the PDBQT format.

Molecular docking calculations were performed using AutoDock Vina (version 1.2.7) [52,53]. The docking search space was defined using a grid box centered at x = 43.90, y = 27.55, and z = 318.18, with dimensions of 24 × 24 × 24 Å. The exhaustiveness parameter was set to 16, and up to 20 binding modes were generated for each ligand. The docking calculations were performed using the same receptor structure and search space for all tested compounds to facilitate comparison of the predicted binding modes and docking scores.

To assess the reproducibility of the docking results, independent docking calculations were performed for CBD, TMZ, and MTIC using different random seeds. The consistency of the resulting docking scores and predicted ligand poses was subsequently evaluated. The predicted binding modes were analyzed based on the docking scores, spatial orientation of the ligands within the receptor binding site, and the amino acid residues located in the vicinity of the docked compounds.

As a supplementary procedural check, the co-crystallized reference ligand AM6538 (identified as ZDG in the PDB structure) was removed from the CB_1_ binding site before docking. The ligand was subsequently redocked into the prepared receptor structure using the applied docking protocol. This approach was used to provide an additional assessment of whether the docking procedure could reproduce a plausible binding mode of a ligand with an experimentally determined position in the CB_1_ binding pocket. The redocking analysis was considered a supplementary procedural check rather than a comprehensive validation of the docking protocol.

### 4.7. Wound Healing (Scratch) Assay

Cell migration was assessed using a wound healing (scratch) assay for the KELLY, SF295, U-87MG, T98G, and C6 cell lines. Cells were seeded in 6-well plates (NEST Biotechnology, Wuxi, China; Catalog #:703001) and cultured until reaching approximately 90–100% confluence. A linear scratch was then made across the cell monolayer using a sterile pipette tip. Following scratching, the wells were gently washed with phosphate-buffered saline (PBS) to remove detached cells and cellular debris. The culture medium was replaced with serum- and antibiotic-free medium containing the tested compound. Images of the scratched areas were captured immediately after scratching (0 h) and after 24 h using an inverted microscope (Nikon Eclipse TS2, Nikon, Tokyo, Japan) equipped with a DeltaPix camera (DeltaPix, Smoerum, Denmark) and imaging software at ×4 magnification. The 24 h period was considered the duration of the experiment. The extent of wound closure was subsequently analyzed by comparing the wound area at 0 and 24 h. The wound healing assay was not performed for the CHP-134 cell line due to its low adherence to the culture surface. The PBS washing step required by the protocol could have caused substantial cell detachment, preventing the formation and maintenance of a uniform cell monolayer and consequently precluding reliable assessment of cell migration.

### 4.8. Western Blot Analysis

The tested cell lines were placed in 6-well plates (NEST Biotechnology, Wuxi, China; Catalog #: 703001); on the following day, the culture medium was removed, and the cells were exposed to cannabidiol at its IC_50_ values for 72 h. After treatment, the cells were harvested, washed twice in ice-cold PBS, lysed in Radioimmunoprecipitation Assay (RIPA) buffer (Sigma-Aldrich, St. Louis, MO, USA; Catalog #:R0278) supplemented with protease inhibitor cocktail (Sigma-Aldrich, St. Louis, MO, USA; Catalog #:539131), and centrifuged at 14,000× *g* for 10 min at 4 °C, as documented earlier [21]. Total protein concentrations in samples were determined using BCA Protein Assay Kit by spectrophotometric analysis following the manufacturer’s instructions (Thermo Scientific, Waltham, MA, USA; Catalog #: A55864). Each sample was diluted with Laemmli solution (containing 5% β-mercaptoethanol) and denatured at 100 °C for 5 min in a thermoblock. Then, equal amounts of the protein lysates were electrophoresed on SDS-PAGE and electroblotted to polyvinyl difluoride (PVDF) membranes (Bio-Rad Laboratories, Hercules, CA, USA). Next, the membranes were blocked in Tris-buffered saline (TBS) with 5% non-fat dry milk and 0.05% Tween 20, pH 7.5, for 2 h at room temperature. Then, the membranes were incubated with primary antibody for 2 h at room temperature: p21, p18, cyclin D1, Bax and Bcl-2 (Cell Signaling Technology, Beverly, MA, USA) and cannabinoid CB_1_ receptor (Invitrogen, Waltham, MA, USA) for glioblastoma (U-87MG and SF295) cell lines. Antibody dilutions were prepared in 5% (*w*/*v*) bovine serum albumin (BSA) according to the product data sheet. After incubation, the membrane was rinsed twice in Tris-buffered saline with 0.01% (*v*/*v*) Tween (TBST) for 10 min. Next, the membrane was treated with a horseradish peroxidase-labeled secondary antibody (Cell Signaling Technology, Beverly, MA, USA) for 1 h at room temperature. After incubation, the membrane was rinsed twice again in Tris-buffered saline with 0.01% (*v*/*v*) Tween (TBST) for 10 min. Finally, the membrane was visualized using a Pierce ECL Western Blotting Substrate (Thermo Scientific, Waltham, MA, USA) according to the manufacturer’s protocol and using Fusion FX (Vilber Lourmat, Marne-la-Vallée, France). After detection of the target protein, the membrane was stripped using Re-Blot Plus Strong Solution (Merck, Darmstadt, Germany, Catalog #:2504) for 15 min, according to the manufacturer’s instructions, thoroughly washed, re-blocked, and subsequently re-probed with an anti-β-actin antibody. Densitometric measurement of protein levels was performed using FusionCapt Advance (ver. FX7 16.12) software. The Western blot was performed independently in duplicate. Densitometric values were averaged from the independent experiments and normalized to the corresponding β-actin expression obtained from the same membrane following the stripping and reprobing procedure. Primary antibodies were used according to the manufacturer’s recommendations (Antibody from Cell Signaling (Beverly, MA, USA): p21 Waf1/Cip1 (12D1) Rabbit Monoclonal Antibody #2947; p18 INK4C (DCS118) Mouse Monoclonal Antibody #2896; cyclin D1 (92G2) Rabbit Monoclonal Antibody #2978; beta-Actin Antibody #4967; Bax (E4U1V) Rabbit Monoclonal Antibody #41162; Bcl-2 (124) Mouse Monoclonal Antibody #15071; antibody from Invitrogen (Waltham, MA, USA): cannabinoid CB_1_ receptor: #PA1-743). Total cellular protein lysates were used for analysis without subcellular fractionation.

### 4.9. Statistical Analysis

The IC_50mix_ and IC_50add_ values for the respective two-drug combinations of cannabidiol with temozolomide (from the log-probit isobolographic analysis) were statistically compared with the unpaired Student’s *t*-test with Welch’s correction, as suggested earlier [31]. The results from Western blot were analyzed with Student’s *t*-test. All statistical tests were calculated with GraphPad Prism (version 8.0, San Diego, CA, USA).

## 5. Conclusions

All the antagonistic interactions determined experimentally for the combination of cannabidiol with temozolomide in various neuroblastoma and glioblastoma cell lines in the MTT assay should not be recommended for clinical practice due to the reduction in the anti-viability effects of the tested drugs in combinations. Both the log-probit isobolographic analysis and the Bliss–Loewe consensus model in the Synergy Finder 3.0 could be recommended for application in in vitro studies to classify the interactions during the development and discovery of novel oncologic therapies. However, the log-probit isobolographic analysis is preferentially recommended for professional researchers, while the Bliss–Loewe consensus model in the Synergy Finder 3.0 is recommended as a screen model during the assessment of interactions for two-drug combinations.

## Figures and Tables

**Table 1:** The anti-viability effects of cannabidiol and temozolomide on neuroblastoma and glioblastoma cell lines in the MTT assay.

	Synergy Finder 3.0		Log-Probit Method	
Cell Line	Cannabidiol µg/mL	Temozolomide µg/mL	Cannabidiol µg/mL	Temozolomide µg/mL
CHP-134	5.74	47.00	5.78 ± 0.72 (72)	42.45 ± 4.75 (72)
KELLY	3.89	38.89	4.11 ± 1.88 (72)	41.58 ± 7.20 (72)
SF295	3.92	295.16	4.02 ± 0.99 (72)	303.90 ± 106.04 (48)
U-87MG	3.85	222.88	3.16 ± 0.49 (72)	219.11 ± 105.28 (72)
T98G	5.76	216.70	5.44 ± 0.57 (48)	213.53 ± 98.94 (72)
C6	4.95	75.86	4.56 ± 1.87 (72)	93.17 ± 35.42 (72)

The results are presented as IC_50_ values (expressed in µg/mL) ± SE (*n*) of the tested drugs determined in experiments performed in triplicates (in both the Synergy Finder 3.0 and log-probit methods).

**Table 2:** Isobolographic analysis of interactions of cannabidiol with temozolomide (at the fixed ratio of 1:1) for parallel concentration–response effects in various neuroblastoma and glioblastoma cell lines in the MTT assay.

Cell Line	IC_50mix_ (*n*_mix_) [µg/mL]	IC_50add_ (*n*_add_) [µg/mL]	Student’s *t*-Test	Interaction
CHP-134	39.96 ± 3.29 *** (24)	24.12 ± 1.58 (140)	t_162_ = 3.907; *p* = 0.0001	Antagonistic
KELLY	46.49 ± 6.57 **** (72)	22.84 ± 2.62 (140)	t_210_ = 3.971; *p* < 0.0001	Antagonistic
SF295	247.01 ± 48.59 (72)	153.96 ± 30.90 (116)	t_186_ = 1.698; *p* = 0.091	Additivity
C6	77.69 ± 28.04 (72)	48.87 ± 10.76 (140)	t_210_ = 1.150; *p* = 0.252	Additivity

The IC_50_ values are presented as the means ± SE (*n*); where IC_50mix_—experimentally derived IC_50_; *n*_mix_—number of samples for experimental mixture; IC_50add_—theoretically additive IC_50_; *n*_add_—number of samples calculated for the additive mixture. Statistical analysis of data was performed with the unpaired Student’s *t*-test. Asterisks: *** and **** reflect significance at *p* < 0.001 and *p* < 0.0001 vs. the respective IC_50add_ value. The last column indicates the type of interaction for the two-drug mixture.

**Table 3:** Isobolographic analysis of interactions of cannabidiol with temozolomide (at the fixed ratio of 1:1) for non-parallel concentration–response effects in various neuroblastoma and glioblastoma cell lines in the MTT assay.

Cell Line	IC_50mix_ (*n*_mix_) [µg/mL]	Lower IC_50add_ (*n*_add_) [µg/mL]	Upper IC_50add_ (*n*_add_) [µg/mL]	Student’s *t*-Test	Interaction
U-87MG	190.31 ± 65.36 (72)	52.27 ± 29.17 (140)	169.99 ± 35.37 (140)	t_210_ = 0.299; *p* = 0.765	Additive
T98G	138.06 ± 29.27 (48)	56.34 ± 28.58 (116)	162.60 ± 32.33 (116)	t_162_ = 0.457; *p* = 0.649	Additive

The IC_50_ values are presented as the means ± SE (*n*), where the IC_50add_ values were calculated from the lower and upper isoboles of additivity; *n*_mix_—total number of samples experimentally determined; *n*_add_—total number of samples calculated for the additive two-drug mixture. Statistical analysis of data was performed with the unpaired Student’s *t*-test. The last column indicates the type of interaction for the two-drug mixture.

**Table 4:** Molecular docking of cannabidiol, temozolomide, and MTIC to the cannabinoid CB_1_ receptor.

Ligand	Docking Run 1 (kcal/mol)	Docking Run 2 (kcal/mol)	Mean Binding Affinity (kcal/mol)	Main Interacting Amino Acid Residues	Predicted Potential Hydrogen-Bond Interactions
Cannabidiol	−8.15	−8.15	−8.15	Phe102, Ser383, Ser199, Gly166, Met103, Ile105, Leu387, Phe268	None detected by the ChimeraX hydrogen-bond analysis
Temozolomide	−6.07	−6.07	−6.07	Ser383, Gly166, Phe170, Val196, Leu387, Cys386	Ser383
MTIC	−5.50	−5.51	−5.50	Cys386, Gly166, Ser383, Phe170, Leu387, Ser390, Trp356	Cys386 and Gly166

Binding affinities correspond to the best-ranked docking pose (mode 1) obtained in each independent docking run. Interacting residues were identified based on spatial contacts within approximately 3.5 Å of the docked ligand. Hydrogen-bond interactions represent computational predictions and were not experimentally confirmed.

**Table 5:** Classification of interactions reported in various pharmacodynamic models when testing the combination of cannabidiol with temozolomide in various neuroblastoma and glioblastoma cell lines in the MTT assay by means of the classic isobolographic analysis and Synergy Finder 3.0 web-application.

Cell Line	Zero Interaction Potential	Bliss	Loewe	Highest Single Agent	Bliss–Loewe	Classic Isobolographic Analysis
CHP-134	Additivity	Additivity	Synergy	Synergy	Additivity	Antagonism
KELLY	Antagonism	Antagonism	Additivity	Additivity	Antagonism	Antagonism
SF295	Additivity	Antagonism	Antagonism	Additivity	Antagonism	Additivity
U-87MG	Additivity	Additivity	Additivity	Additivity	Antagonism	Additivity
T98G	Additivity	Antagonism	Antagonism	Additivity	Antagonism	Additivity
C6	Antagonism	Antagonism	Additivity	Antagonism	Antagonism	Additivity

## Data Availability

Data are contained within the article. Supporting Information is available in the additional Supplementary Files, and further supporting data is available from the authors on request.

## Supplementary Materials

The following supporting information can be downloaded at https://www.mdpi.com/article/10.3390/ijms27219946/s1.

## Abbreviations

The following abbreviations are used in this manuscript:

CB_1_	Cannabinoid type 1 receptor
CBD	Cannabidiol
HSA	Highest Single Agent
MTIC	5-(3-methyltriazen-1-yl)imidazole-4-carboxamide
MTT	3-(4,5-dimethylthiazol-2-yl)-2,5-diphenyltetrazolium bromide
TMZ	Temozolomide
ZIP	Zero Interaction Potential
