# Supplementary material for: Detection of Antagonistic Interactions Between Cannabidiol and Temozolomide in Various Neuroblastoma and Glioblastoma Cell Lines in the MTT Assay by Means of Synergy Finder 3.0 Software and Log-Probit Isobolographic Analysis
Source: Int J Mol Sci. 2026 Nov 6;27(21):9946. doi: 10.3390/ijms27219946 (PMC13763955; doi:10.3390/ijms27219946)
Supplement: Supplementary file 1 [file ijms-27-09946-s001.zip › ijms-4626537-supplementary.pdf]

Supplementary Materials

Detection of antagonistic interactions between cannabidiol and temozolomide in various neuroblastoma and glioblastoma cell lines in the MTT assay by means of Synergy Finder 3.0 software and log-probit isobolographic analysis

Katarzyna Załuska-Ogryzek [1], Paula Wróblewska-Łuczka [1], Agnieszka Góralczyk[1] and Jarogniew J. Łuszczki [1,*]

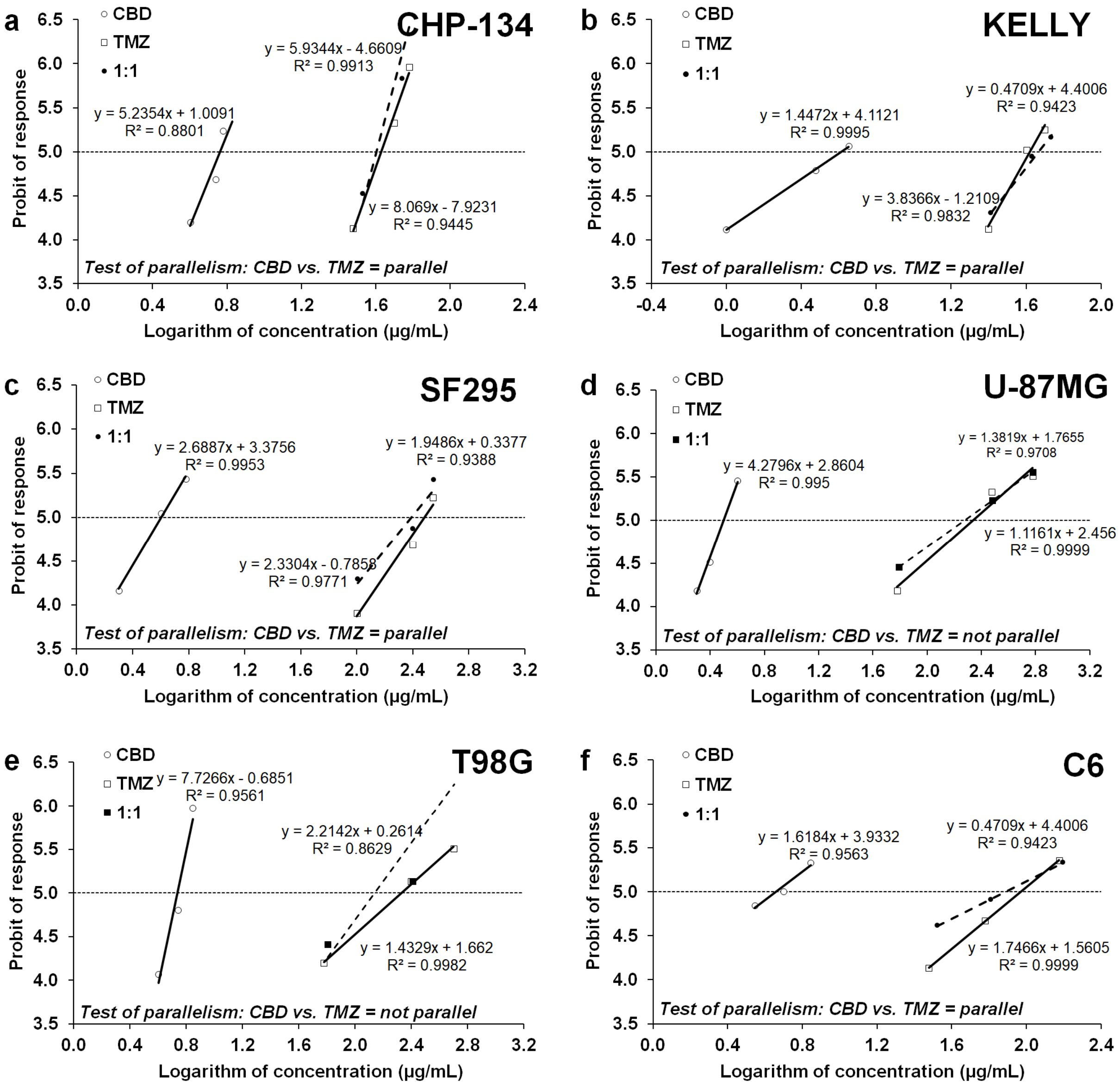

Figure S1. Log-probit concentration–response effect lines for cannabidiol, temozolomide and their combination in the MTT assay. Collateral and non-parallel lines display the anti-viability effects of the tested drugs in various neuroblastoma and glioblastoma cell lines: CHP-134 (a), KELLY (b), SF295 (c), U-87MG (d), T98G (e), and C6 (f) measured in the MTT assay.

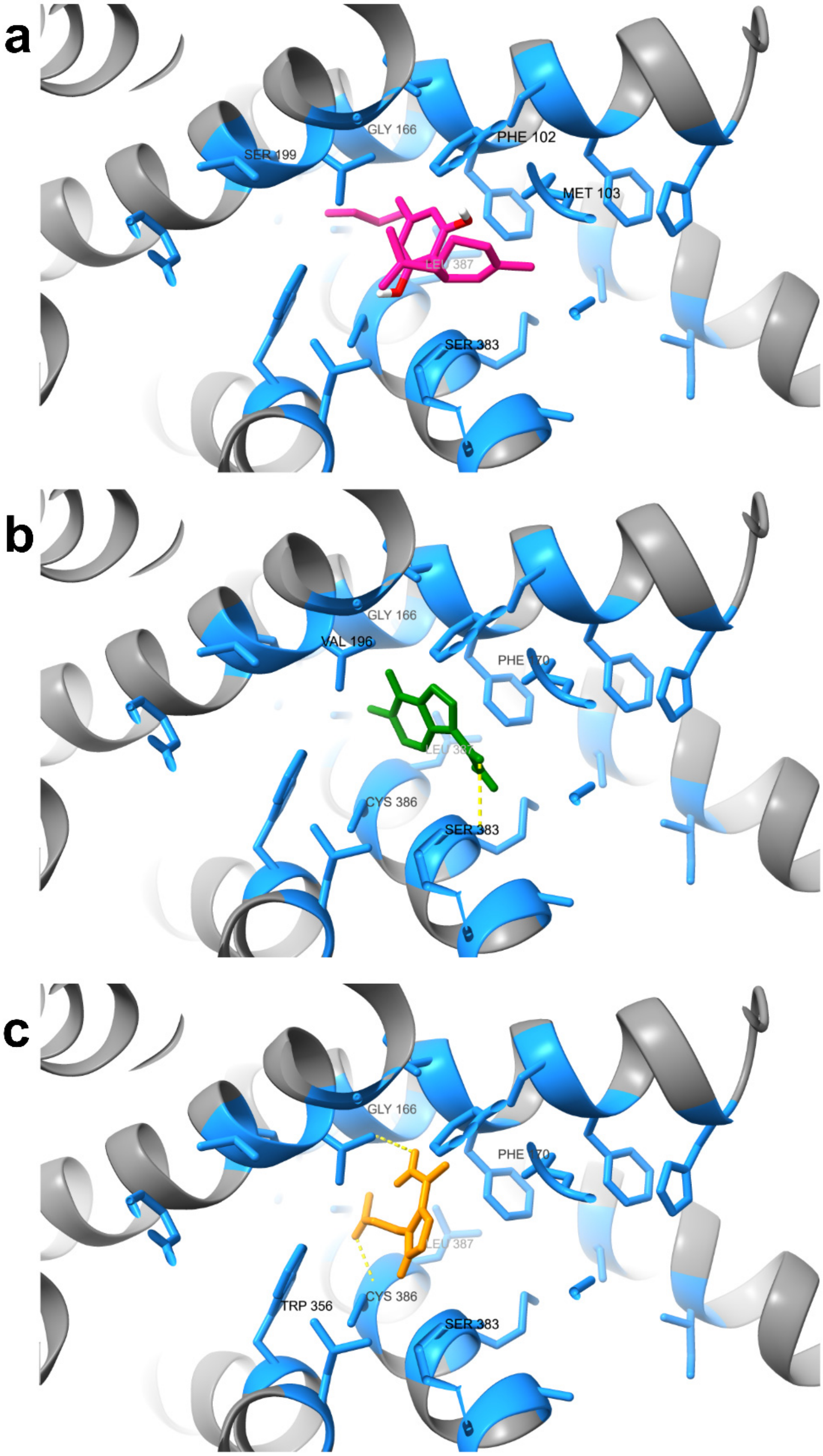

Figure S2. Predicted binding modes of cannabidiol, temozolomide, and MTIC within the cannabinoid CB_1 receptor binding pocket. Molecular docking poses of (a) cannabidiol (pink), (b) temozolomide (green), and (c)

MTIC (orange) within the cannabinoid CB_1 receptor binding site. The receptor is shown as a gray ribbon, while selected residues surrounding the docked ligands are presented as blue sticks and labeled according to their residue type and number. Yellow dashed lines indicate predicted hydrogen-bond interactions identified using the ChimeraX hbonds tool. A potential hydrogen-bond-like interaction was identified between temozolomide and Ser383, whereas two predicted hydrogen bonds were detected between MTIC and Gly166 and Cys386. No hydrogen bonds were identified for cannabidiol under the applied docking analysis criteria. The molecular representations were generated using UCSF ChimeraX. The same receptor orientation was maintained across all panels to facilitate visual comparison of the predicted ligand-binding modes.

Table S1. Detailed docking results and predicted interactions of cannabidiol, temozolomide, and MTIC with the cannabinoid CB_1 receptor in two independent docking runs.

Ligand	Docking run	Best binding affinity (kcal/mol)	Predicted interacting residues	Potential hydrogen-bond interactions
cannabidiol	Run 1	−8.149	Phe102, Ser383, Ser199, Gly166, Met103, Ile105, Leu387, Phe268	None detected
cannabidiol	Run 2	−8.153	Ser383, Phe102, Ser390, Ile105, Gly166, Cys386, Leu387, Val196, Met103, Leu359	None detected
temozolomide	Run 1	−6.066	Ser383, Gly166, Phe170, Val196, Leu387, Cys386	Ser383; 3.262 Å (D···A)
temozolomide	Run 2	−6.072	Ser383, Gly166, Phe170, Val196, Leu387, Cys386	Ser383; 3.230 Å (D···A)
MTIC	Run 1	−5.501	Cys386, Gly166, Ser383, Phe170, Leu387, Ser390, Trp356	Cys386: 3.021 Å (D···A), 2.133 Å (D–H···A); Gly166: 3.171 Å (D···A), 2.344 Å (D–H···A)
MTIC	Run 2	−5.507	Cys386, Gly166, Ser383, Phe170, Leu387, Ser390, Trp356	Cys386: 3.020 Å (D···A), 2.125 Å (D–H···A); Gly166: 3.158 Å (D···A), 2.332 Å (D–H···A)

The reported binding affinity corresponds to mode 1 from each independent docking run. Interacting residues were identified using ChimeraX contact analysis with a distance cutoff of approximately 3.5 Å. Potential hydrogen-bond interactions were identified using the ChimeraX hydrogen-bond analysis and should be interpreted as model-based predictions. For temozolomide, the reported interaction with Ser383 was classified as hydrogen-bond-like because the complete conventional hydrogen-bond geometry was not established. For MTIC, potential interactions were identified with Cys386 and Gly166.

D···A denotes the donor–acceptor distance, whereas the second distance corresponds to the hydrogen-related distance reported by ChimeraX. The identified interactions are computational predictions and should not be considered experimentally confirmed hydrogen bonds.
